# Small Rad51 and Dmc1 Complexes Often Co-occupy Both Ends of a Meiotic DNA Double Strand Break

**DOI:** 10.1371/journal.pgen.1005653

**Published:** 2015-12-31

**Authors:** M. Scott Brown, Jennifer Grubb, Annie Zhang, Michael J. Rust, Douglas K. Bishop

**Affiliations:** 1 Department of Molecular Genetics and Cell Biology, University of Chicago, Cummings Life Science Center, Chicago, Illinois, United States of America; 2 Department of Radiation and Cellular Oncology, University of Chicago, Cummings Life Science Center, Chicago, Illinois, United States of America; National Cancer Institute, UNITED STATES

## Abstract

The Eukaryotic RecA-like proteins Rad51 and Dmc1 cooperate during meiosis to promote recombination between homologous chromosomes by repairing programmed DNA double strand breaks (DSBs). Previous studies showed that Rad51 and Dmc1 form partially overlapping co-foci. Here we show these Rad51-Dmc1 co-foci are often arranged in pairs separated by distances of up to 400 nm. Paired co-foci remain prevalent when DSBs are dramatically reduced or when strand exchange or synapsis is blocked. Super-resolution dSTORM microscopy reveals that individual foci observed by conventional light microscopy are often composed of two or more substructures. The data support a model in which the two tracts of ssDNA formed by a single DSB separate from one another by distances of up to 400 nm, with both tracts often bound by one or more short (about 100 nt) Rad51 filaments and also by one or more short Dmc1 filaments.

## Author Summary

During meiosis, a specialized form of chromosome segregation ensures that gametes contain only one copy of the parental chromosome complement. Accurate segregation of maternal and paternal chromosomes requires them to first become connected in pairs. Homologous recombination forms these needed connections. Connections between homolog chromosomes are made by forming and then repairing DNA double strand breaks. Rad51 and Dmc1 are structurally related enzymes that form complexes by binding DNA at sites of breaks, where they then function to promote break repair by searching for and invading corresponding unbroken DNA sequences on a homologous chromosome. In this paper, we describe several features of the recombination complex structure. We provide evidence that: 1. both Rad51 and Dmc1 load onto both ends of a single DSB, accounting for the known activity of both ends of a DSB; 2. the two ends of a DSB can separate by distances of up to 0.4 microns; 3. Rad51 and Dmc1 complexes only occupy small segments of DNA (about 100 bases); 4. multiple short Rad51 and Dmc1 complexes can occupy a single DSB end.

## Introduction

Meiotic recombination is a highly regulated process that faithfully repairs programed DSBs, ensuring accurate reductional chromosome segregation at meiosis I[1]. Following pre-meiotic DNA replication, Spo11 introduces DSBs across the genome. These DSBs are nucleolytically resected, revealing 3’ single strand DNA (ssDNA) tracts that are subsequently used to locate an intact, homologous double strand DNA (dsDNA) repair template. Upon completing the homology search, the ssDNA invades the intact dsDNA duplex creating a displacement-loop structure. The invading 3’ end serves as a primer for restorative DNA synthesis, facilitating the completion of the DNA repair process.

During meiosis, the eukaryotic RecA homologs Rad51 and Dmc1 cooperate to promote homology search and strand exchange, the central step in homologous recombination[2]. Like RecA, Rad51 and Dmc1 form nucleoprotein filaments on ssDNA and catalyze strand exchange *in vitro*[3–6]. Rad51 is responsible for catalyzing strand exchange *in vivo* in mitotically cycling cells[7]. However, the meiosis-specific protein Dmc1 is the predominant meiotic strand exchange enzyme[8–10]. Rad51’s activity is inhibited during meiosis by the Hed1 protein[11,12]. Nonetheless, Rad51 plays an important non-enzymatic role, promoting Dmc1 assembly and directing it to invade a homolog chromatid rather than a sister chromatid[9,13–15].

Rad51 and Dmc1 form spatially associated repair complexes in accord with their genetic interaction. In spread meiotic *S*. *cerevisiae* nuclei, Rad51 and Dmc1 form DSB-dependent foci[16]. Rad51 and Dmc1 approximately co-localize as side-by-side, partially offset “co-foci” when viewed by widefield epifluoresence microscopy [16–18]. Combined with the propensity of Rad51 and Dmc1 to interact homotypically but not heterotypically[19–21], this staining pattern led to speculation that Rad51 and Dmc1 homofilaments might occupy opposite ends of each DSB[17,18]. This speculation, along with a number of other observations, influenced the development of models of meiotic recombination involving asymmetric loading of Rad51 and Dmc1 on opposite DSB ends[1,15].

The asymmetric loading model in which Rad51 and Dmc1 homofilaments occupy opposite ends of a single DSB has awkward implications. First, the model implies that Rad51 is selectively loaded onto one member of a pair of ends, somehow avoiding the other end of each DSB. Second, Rad51 is required for normal assembly of Dmc1[16,22], thus the asymmetric loading model requires Rad51 to accomplish this assembly function *in trans* on the opposite DSB ssDNA tract. Third, the asymmetric loading model implies that only the Dmc1-decorated end is capable of strand exchange, given the disposability of Rad51’s catalytic activity[9–11]. While the asymmetric loading model has been argued to account for the apparent differentiation of the two ends of a DSB[1,15], both ends of a DSB can catalyze strand exchange as evidenced by multichromatid joint molecules that are normally disassembled by the Sgs1 helicase[23]. Here we present evidence contradicting the asymmetric loading model and supporting a model in which both Rad51 and Dmc1 can and often do load on both DSB ends. Additionally, we demonstrate that Rad51 and Dmc1 filaments are very short *in vivo* coating only about 100 nt of ssDNA.

## Results

### Rad51-Dmc1 Co-foci Form Pairs that Can Be Separated by up to 400 nm

**Fig 1:**
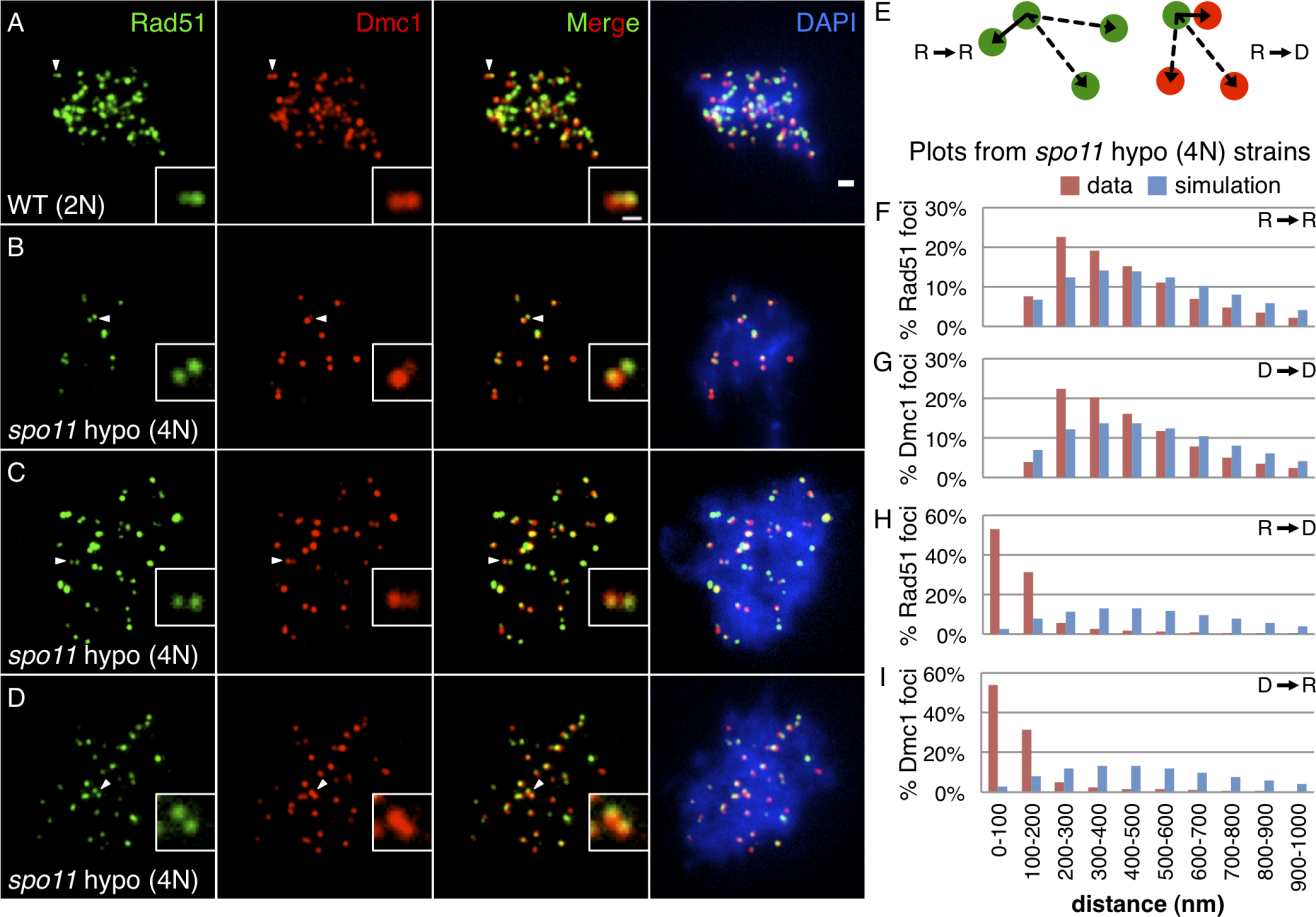
Rad51-Dmc1 co-foci occur in pairs separated by 200–400 nm. **(A)** Spread wild type diploid [WT (2N)] nucleus stained for Rad51 (green) and Dmc1 (red). **(B,C,D)** Spread *spo11* hypomorphic tetraploid [*spo11* hypo (4N)] nuclei with relatively low densities of Rad51-Dmc1 staining. Arrowheads indicate examples of paired co-foci in less densely stained areas, magnified in the insets. Scale bar = 1 μm, 400 nm for the inset. **(E)** Schematic of distance to nearest neighbor measurement for Rad51-to-Rad51 (left) and Rad51-to-Dmc1 (right). Solid arrow represents the distance to the reference focus’ nearest neighbor. Dashed arrows represent the longer distances to other foci. **(F,G,H,I)** Distribution of nearest neighbor measurements in wild type *spo11* hypomorphic tetraploid. Experimental data (red) and matched random simulations (blue) shown for **(F)** Rad51→Rad51, **(G)** Dmc1→Dmc1, **(H)** Rad51→Dmc1, and **(I)** Dmc1→Rad51. Micrographs from cultures 2.5 hours after meiotic induction. Scoring of 195 focus-positive nuclei, of all staining densities, containing a total of 13,528 Rad51 foci and 13,230 Dmc1 foci are included in the histograms.

When viewed by widefield microscopy, a substantial fraction of Rad51-Dmc1 co-foci appear to be arranged in pairs separated by up to 400 nm in sparsely populated regions of meiotic *S*. *cerevisiae* spread diploid (2N) nuclei (Fig 1A, arrowhead). It was unclear if this apparent pairing might simply be due to the fortuitous arrangement of unrelated foci in a crowded nucleus. To reduce the potential bias imposed by crowding, we constructed a tetraploid (4N) strain hypomorphic for *SPO11*. This tetraploid carries one wild type (*SPO11* ^*+*^) allele and three alleles that code catalytically inactive protein (*spo11-Y135F*) [24]. Spo11 is the transesterase that forms DSBs[25,26]. As expected for a strain with reduced DSB levels and larger nuclei, the average density of Rad51 (and Dmc1) foci per unit area was lower in the *spo11* hypomorphic tetraploid than in wild type diploids. However, individual nuclei with focus densities ranging from very low to the same as that in wild type diploid nuclei were observed, likely reflecting the homeostatic control of DSB levels that eventually compensate for reduced Spo11 activity[14,27–29]. When the analysis was restricted to tetraploid nuclei early in prophase (2.5 hr) with low densities of foci, pairs of foci separated by ≤ 400 nm could clearly be seen (Fig 1B–1D). Thus, Rad51-Dmc1 co-foci tend to be arranged in pairs.

To quantitatively assess the prevalence of the paired co-focus architecture, the distance between each focus and its nearest neighboring focus was measured in the *spo11* hypomorphic tetraploid strains. For example, the shortest distance between the centroid of a Rad51 focus and the centroid of the nearest neighboring Rad51 focus was determined (solid lined arrow, Fig 1E, left). This process was repeated for each Rad51 focus in the nucleus, and such measurements from many nuclei were pooled to form a Rad51-to-Rad51 (R→R) nearest neighbor distribution (Fig 1F, red). The limited resolution of light microscopy makes it difficult to distinguish foci separated by distances below 200 nm, resulting in estimates of inter-focus distances that decay below 200 nm with a minimum inter-focus distance of about 150 nm in this assay (see Analysis section of Materials and Methods). To evaluate the likelihood that this distribution could be generated by the independent, random assortment of individual foci, a simulated Rad51-to-Rad51 nearest neighbor distribution was generated by randomly positioning foci within a nuclear area (Fig 1F, blue, see Methods). For a given distance bin, a higher frequency of experimental pairs than of simulated pairs indicates that such pairs are enriched relative to the expectation based on the random arrangement of foci.

Quantitative analysis of nearest neighbor distributions confirmed that both Rad51 and Dmc1 foci are non-randomly arranged in pairs separated by up to 400 nm (Fig 1F and 1G). This enrichment of focus pairs separated by ≤400 nm was detected in both unselected nuclei and nuclei selected for low focus density (S1A–S1D Fig). Additionally, paired foci are not simply due to local crowding, since ≤400 nm pairs are enriched in locally sparse regions (S1E and S1F Fig). This analysis cannot make strong conclusions regarding the prevalence of pairing below 200 nm (due to the diffraction limit of light). The method also has limited power to identify non-random spatial patterns at distances greater than 400 nm, because it reports only on the closest distance between structures. However, if a precise longer pairing distance were present, it could not account for more than 15% of Rad51 or Dmc1 foci because a subpopulation larger than that size would have been detected (Fig 1F and 1G). We also used the nearest neighbor methodology to assess the spatial relationship of Rad51 foci to Dmc1 foci and vice versa (Fig 1E, right). 84% of Rad51 foci are less than 200 nm from the nearest neighboring Dmc1 focus (Fig 1H). Similarly, 85% of Dmc1 foci are less than 200 nm from a Rad51 focus (Fig 1I). It should be noted that this analysis differs from that of measuring distances between resolved pairs of Rad51, or resolved pairs of Dmc1 foci, because the use of different fluorophores to detect Rad51 and Dmc1 allows measurement of nearest neighbor distances below the resolution limit. The distributions of Rad51-Dmc1 distances support the longstanding observation that Rad51 and Dmc1 foci colocalize imperfectly in a side-by-side configuration[16–18]. In the context of a ≤400 nm pair, each focus in a pair could be a Rad51-Dmc1 co-focus, a Rad51-only focus, or a Dmc1-only focus. All permutations of pair composition were observed with no apparent difference between Rad51 and Dmc1 (e.g. a Rad51 focus was paired with a co-focus equally often as a Dmc1 focus was paired with a co-focus). A conservative measurement indicates that at least 50% of all focus pairs contain two Rad51-Dmc1 co-foci. Furthermore, the staining intensity of Rad51 or Dmc1 within one half of a co-focus pair does not predict the staining intensity of Rad51 or Dmc1 within the other half of the co-focus pair (S1G–S1J Fig). These results suggest each Rad51-Dmc1 co-focus assembles independently of its partner co-focus.

### Pairs of Rad51-Dmc1 Co-foci Form at Individual Meiotic DSBs

**Fig 2:**
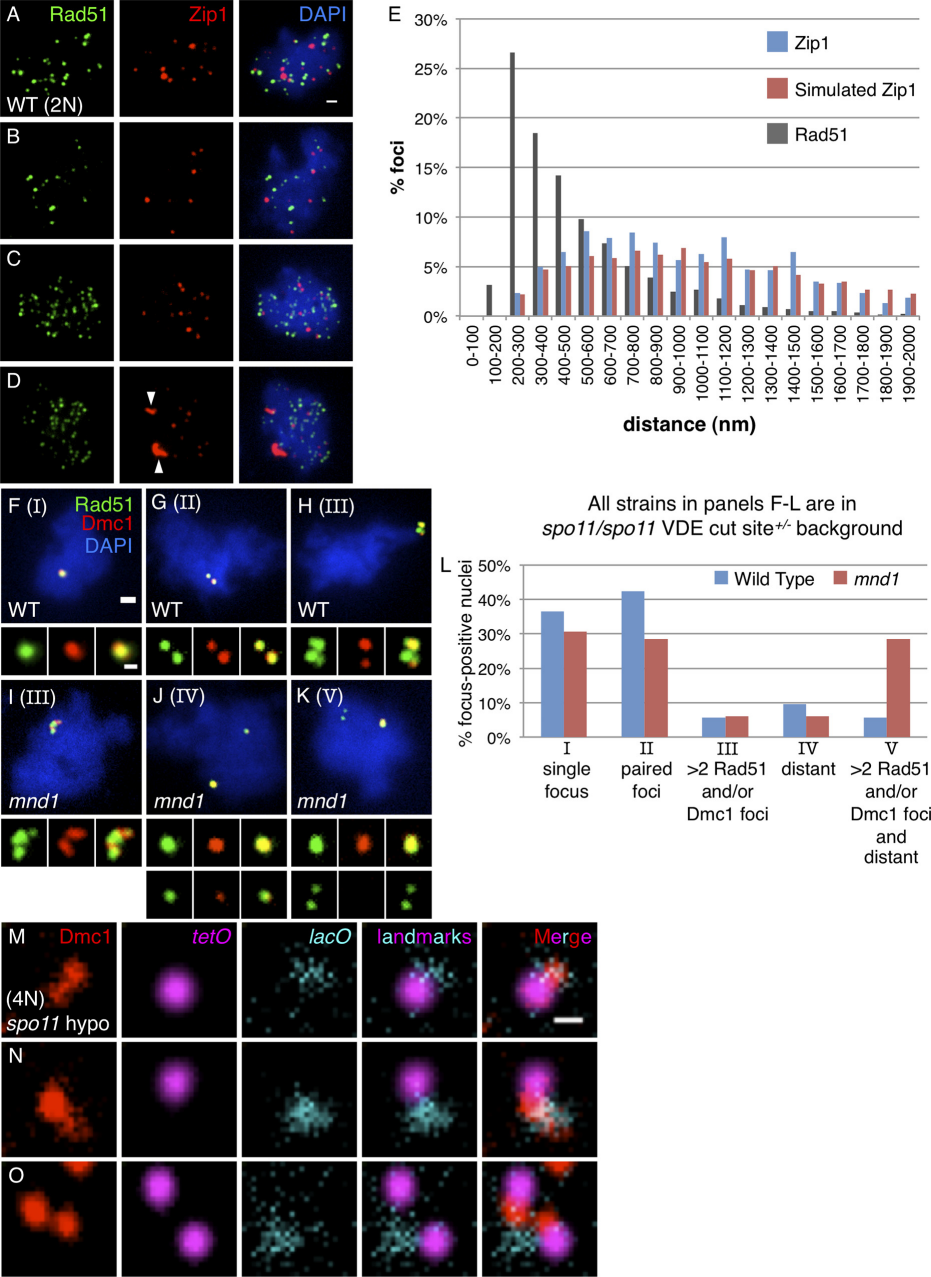
Rad51 and Dmc1 form structures inconsistent with asymmetric loading at individual meiotic DSBs. (**A-E**) Rad51 focus pairing is distinct from the spatial arrangement of Zip1. (**A-C**) Micrographs of wild type diploid leptotene (3 hr) nuclei included in the analysis in (E). Rad51 staining is shown in green, Zip1 staining in red, and DAPI in blue. (**D**) Micrograph of nucleus excluded from analysis in (E) because of non-punctate Zip1 structures (arrowheads). (**E**) Nearest neighbor distributions for Zip1 (blue), simulated Zip1 (red), and Rad51 (grey). Sample size is 86 nuclei containing 2,953 Rad51 foci and 1,041 Zip1 foci. Scale bar in panel A is 1 μm. **(F-L)** Characterization of Rad51-Dmc1 structures in *spo11* diploids heterozygous for the VDE cut site. Rad51 staining is shown in green, Dmc1 in red, and DAPI in blue. **(F-H)** Micrographs of wild type nuclei with **(F)** a single focal structure (category I), **(G)** paired foci (category II), and **(H)** a more complicated structure (>2 Rad51 and/or Dmc1 foci; category III; categories shown in parentheses at top left of each micrograph). **(I)** Micrograph of *mnd1* strand exchange mutant with more complicated structures containing >2 Rad51 and/or Dmc1 foci in close proximity (category III). **(J)** Micrograph of spatially distinct structures each composed of a single focus (category IV) in an *mnd1* mutant. **(K)** Micrograph of a nucleus with >2 Rad51 and/or Dmc1 structures that are also spatially separated (category V) in an *mnd1* mutant. Scale bar = 1 μm, 400 nm for inset. **(L)** Distribution of these structures in wild type and *mnd1* nuclei represented as a percentage of focus-positive nuclei. Micrographs from cultures 4.5 hours after meiotic induction. 116 wild type and 89 *mnd1* focus-positive nuclei are included. **(M-O)** Paired Dmc1 foci can be observed at the *HIS4*::*LEU2* DSB hotspot. Micrographs of paired Dmc1 foci near the *lacO* (*ARS 308*) and *tetO* (*ARS 304*) landmarks flanking the *HIS4*::*LEU2* DSB hotspot. Dmc1 is shown in Red, *tetO* in magenta, and *lacO* in cyan. Scale bar = 500 nm. **(O)** Micrograph in which sister chromatids are split as indicated by a pair of *tetO* foci. Micrographs from cultures 2.5 hours after meiotic induction.

The enrichment of Rad51 (and Dmc1) pairing at distances ≤400 nm is not simply explained by the restriction of foci to positions along an underlying, but invisible, linear chromosome structure. In leptotene nuclei, Zip1 foci form along the otherwise invisible, linear chromosome axis. Yet, the Zip1 nearest neighbor distribution peaks at longer distances than the Rad51 distribution measured in the same nuclei (Fig 2A–2E). Furthermore, the Zip1 nearest neighbor distribution resembles the distribution expected from a random arrangement of Zip1 foci (Fig 2E). These observations are consistent with the possibility that individual pairs of Rad51-Dmc1 co-foci represent meiotic recombination complexes associated with a single DSB.

Nuclei with a single DSB site provided evidence that each DSB often gives rise to a pair of foci. To further rule out the possibility that paired Rad51-Dmc1 co-foci represent adjacent DSBs along a single chromosome, recombination complexes were characterized in a *spo11* strain heterozygous for the VDE cut site. This background is heterozygous for a cleavage site for the meiosis-specific endonuclease VDE on *chromosome IV*. During meiotic prophase, both sister chromatids are eventually cleaved and repaired via recombination with a homolog chromatid, resulting in about 86% 4:0 gene conversions[30,31]. The kinetics of cleavage and repair at the VDE cut site have not (to our knowledge) been assayed in single cells, but at any given time 0, 1, or 2 DSBs are expected to be present. In chromosome spread preparations, VDE-dependent Rad51-Dmc1 foci were readily observed. While only 4% (2/47) of *spo11* nuclei were focus-positive, 47% (54/116) of wild type and 56% (49/88) of *mnd1* nuclei were focus-positive in the *spo11* VDE cut site heterozygote background. Nuclei with a single focus or a single pair of foci are the predominant classes observed in wild type cells (Fig 2F and 2G and 2L; categories I and II) representing 79% of total focus-positive nuclei. However, a more complex class of structures containing more than two Rad51 foci and/or more than two Dmc1 foci in close proximity to each other was observed in about 6% of focus-positive wild type nuclei (Fig 2H and 2I and 2L; category III). Additionally, nuclei with distant cytological complexes (greater than 1 μm apart) were observed in about 15% of wild type nuclei: 9.6% contained no more than two Rad51 or two Dmc1 foci (Fig 2J and 2L; category IV) and 5.8% contained greater than two Rad51 or two Dmc1 foci (Fig 2K and 2L; category V). Importantly, 11.5% of wild type nuclei with greater than two Rad51 foci or two Dmc1 foci (categories III and V) cannot be explained by the asymmetric loading model in which two Rad51-Dmc1 co-foci—one co-focus at each of two DSBs—is the most complicated predicted structure. Moreover, a model in which both Rad51 and Dmc1 can co-occupy each of the four ssDNA tracts associated with two DSBs readily explains these data. This interpretation is further supported by the accumulation of category III and V nuclei to levels up to 39% of focus-positive cells in *mnd1* mutants defective for strand exchange (Fig 2I and 2K and 2L)[32–34]. We interpret distant cytological complexes as likely reflecting situations in which the VDE sites on both sister chromatids are simultaneously cleaved, breaking the sister chromatid pair entirely, such that the two halves of the broken chromosome separate *in vivo* and/or during the spreading procedure. Supporting this interpretation, the incidence of distantly separated foci was greater among nuclei with more than 2 Rad51 and/or Dmc1 foci (50%) than among nuclei with only two foci (19%). Furthermore, the class of nuclei with distantly separated foci (categories IV and V) was more predominant in an *mnd1* mutant, primarily due to an increase in category V nuclei (Fig 2J and 2K and 2L). This observation suggests that blockage of Mnd1-mediated strand exchange results in the accumulation of DSBs leading to the complete breakage of *chromosome IV*, and loading of Rad51 and/or Dmc1 on all four associated ssDNA tracts. In conclusion, the range of structures observed in *spo11* strains heterozygous for the VDE cut site are best explained if both Rad51 and Dmc1 can load onto both ends of a single meiotic DSB.

Co-focus pairs can be seen at a strong meiotic recombination hotspot. To obtain further evidence that paired Rad51-Dmc1 co-foci form at a single DSB, we simultaneously visualized co-foci and fluorescent landmarks flanking the *HIS4*::*LEU2* DSB hotspot on *chromosome III*. Specifically, tandemly repeated arrays of the bacterial transcription factor binding sites *lacO* and *tetO* were integrated about 60 kb away proximal to the centromere and 37 kb away distal to the centromere, respectively, relative to the *HIS4*::*LEU2* DSB hotspot on *chromosome III*, in the *spo11* hypomorphic tetraploid strain with only a single chromatid bearing the *HIS4*::*LEU2* DSB hotspot. Expression of 3xHA-LacI and YFP-TetR allows the arrays to serve as chromosomal landmarks that can be visualized along with Rad51 or Dmc1 foci via immunostaining. Although the position of the *lacO* array was not always detectable (due to partial proteolysis of the 3xHA-LacI fusion protein as demonstrated cytologically and by western blot), a significant subset of nuclei displayed both landmarks. Considering only those nuclei that display both landmarks and a low density of Dmc1 foci in the vicinity of the landmarks, 41% (16/39) had one resolvable pair of Dmc1 foci at the hotspot (Fig 2M–2O); the remainder had a single focus. Similarly, 39% (19/49) of selected nuclei had paired Rad51. Given previous results showing that breakage of both sister chromatids is very rare at the *HIS4*::*LEU2* hotspot (only 11% of tetrads show evidence of two different recombination events in a less severe *spo11* hypomorph than the one used in this study)[35], the data provide additional evidence that a single DSB produces a co-focus pair.

### Sister Chromatids Are Often Spatially Separated

Interestingly, the *tetO* landmark signal was split in 50% or more of nuclei, indicating separation of sister chromatids 37 kb away from the *HIS4*::*LEU2* DSB hotspot (for example, Fig 2O). Although the frequency of *tetO* splitting increases after meiotic induction, the vast majority of the observed splitting is *SPO11*-independent (76% vs. 67% of total nuclei in wild type and *spo11*, respectively; n = 45 nuclei for each). When there was an optically resolvable pair of split *tetO* spots, they were separated by around 400 nm (381±144; range 240–1008; 94% are between 240 and 600 nm), similar to the distance between paired Rad51 (and Dmc1) foci.

### Rad51-Dmc1 Co-focus Pairing Does Not Require Strand Exchange or Synapsis

**Fig 3:**
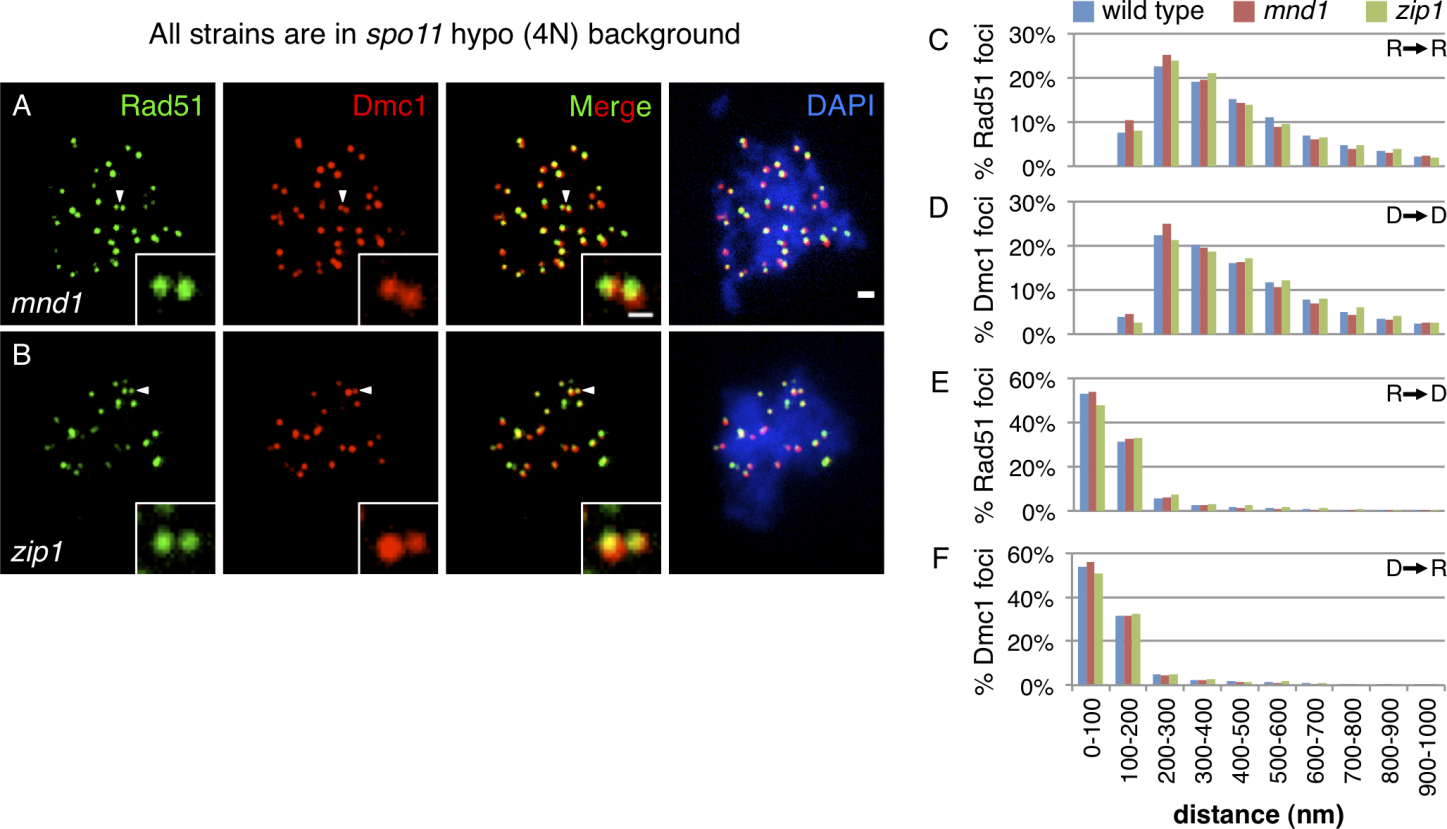
The paired architecture of Rad51-Dmc1 co-foci is independent of strand exchange and synapsis. **(A,B)** Micrographs of **(A)** *mnd1* and **(B)** *zip1* mutant nuclei in the *spo11* hypomorphic tetraploid background. Scale bar = 1 μm, 400 nm for the inset. Rad51 staining is shown in green, Dmc1 in red, and DAPI in blue. **(C-F)** Accompanying distribution of nearest neighbor measurements as in Fig 1 for **(C)** Rad51→Rad51, **(D)** Dmc1→Dmc1, **(E)** Rad51→Dmc1, and **(F)** Dmc1→Rad51. Micrographs from cultures 2.5 hours after meiotic induction. Wild type data repeated from Fig 1 for comparison. Scoring of 70 and 44 focus-positive nuclei; 6,364 and 2,824 Rad51 foci; and 6,114 and 2,655 Dmc1 foci are included in histograms for *mnd1* and *zip1* mutants, respectively.

To probe the relationship between the paired co-focus architecture and both the progression of recombination reactions and transitions in global chromosome structure, nearest neighbor distributions were determined in strand exchange-defective (*mnd1*) and synapsis-defective (*zip1*) mutants. When strand exchange was blocked in an *mnd1* mutant[32–34], the paired character of Rad51 (and Dmc1) foci was maintained (Fig 3A and 3C and 3D). The pairing of Rad51 (and Dmc1) foci was also unaltered in the *zip1* mutant which blocks the progress of recombination reactions after strand exchange (Fig 3B–3D) [36–38]. In *zip1* mutants, nascent post-strand exchange intermediates promote homolog co-alignment at a distance of 400 nm or less, but the closer 100 nm alignment of lateral elements resulting from elongation of the synaptonemal complex does not occur[38]. Together, these mutants suggest that the ≤400 nm paired Rad51-Dmc1 co-focus architecture does not require strand exchange and is retained until synapsis. Further analysis demonstrated that the nearest neighbor distributions of Rad51-to-Dmc1 and Dmc1-to-Rad51 distances are also unaltered in strand exchange-defective and synapsis-defective mutants (Fig 3E and 3F), suggesting that the side-by-side Rad51-Dmc1 configuration is also established prior to, and persists after, strand exchange.

### Small Rad51 and Dmc1 Structures Are Paired at Sub-diffraction Distances

**Fig 4:**
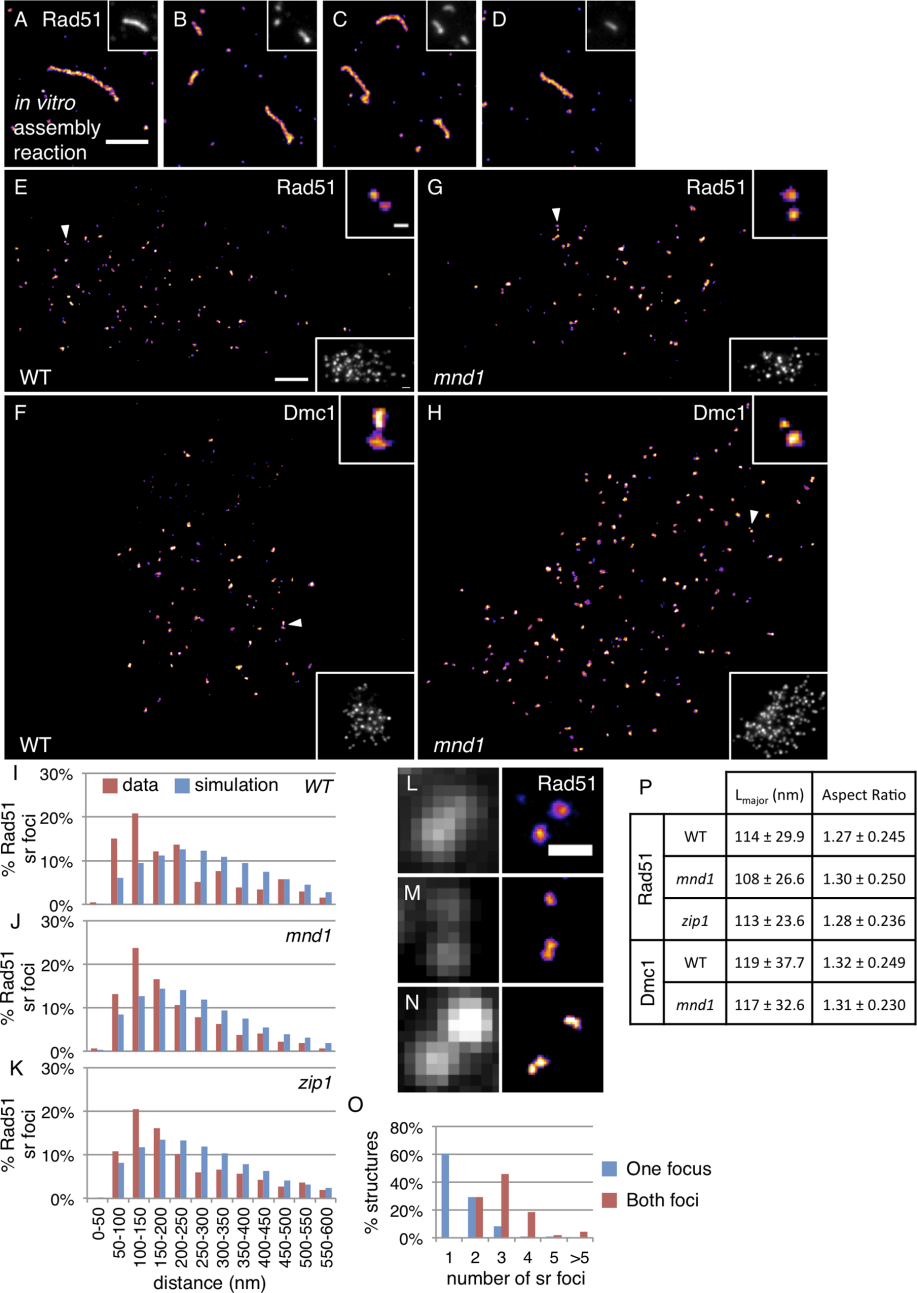
Rad51 and Dmc1 sr foci are extremely small and clustered at sub-diffraction distances. **(A-D)** dSTORM micrographs of Rad51 filaments assembled on a linear 2.7 kbp dsDNA *in vitro* in the presence of the meiotic protein Hed1. Corresponding widefield micrographs are inset at top right and the scale bars are 1 μm wide (**E-H**) Example dSTORM and widefield (bottom right insets) micrographs of wild type nuclei stained for (**E**) Rad51 and (**F**) for Dmc1; *mnd1* nuclei stained for (**G**) Rad51 and (**H**) Dmc1. Arrowheads indicate examples of 100 nm paired Rad51 sr foci, magnified in the upper right insets. Scale bar is 1 μm for dSTORM and widefield micrographs, 100 nm in top right insets. **(I-K)** Distributions of Rad51→Rad51 sr foci nearest neighbor measurements for **(I)** wild type, **(J)** *mnd1*, and **(K)** *zip1* mutants. Micrographs from cultures 3.5 hours after meiotic induction. (**L-O**) Each Rad51 focus in a pair of Rad51 foci imaged under widefield can contain more than one Rad51 sr focus. (**L-N**) Micrographs of paired Rad51 foci observed at widefield resolution (left) and with dSTORM (right) containing 1 and 1 (L); 1 and 2 (M); and 2 and 2 (N) Rad51 sr foci. Scale bar is 400 nm. (**O**) Distribution of the frequencies of Rad51 sr foci in one focus (blue) or both foci (red) in paired Rad51 foci at low resolution. 48 widefield pairs of Rad51 foci were scored. **(P)** Characterization of the dimensions of Rad51 and Dmc1 sr foci. An ellipse was fit to each sr focus. L_major_ is the length of the ellipse’s long axis; aspect ratio equals L_major_/L_minor_. Scoring of 432, 212, and 304 sr foci from 5 wild type, 5 *mnd1*, and 5 *zip1* nuclei for Rad51 scoring; 359 and 592 sr foci from 3 wild type and 6 *mnd1* nuclei for Dmc1 scoring. Errors are S.D.

To characterize recombination complexes in more detail we used direct stochastic optical reconstruction microscopy (dSTORM), a method with higher resolution than standard widefield microscopy[39,40]. We validated our experimental system by imaging long Rad51 filaments prepared by the same method as that used previously for analysis by electron microscopy[41–43]. The Rad51 filaments were assembled on dsDNA *in vitro*, deposited on a coverslip, immunostained, and imaged. As expected, reconstructed dSTORM micrographs clearly show long Rad51 filaments often exceeding one micron in length (Fig 4A–4D). Following indirect immunostaining, the structures observed are 70 nm wide, 60 nm wider than the underlying protein filament as a consequence of both antibody decoration and the finite resolution of the imaging method. Given this apparent filament width, only filaments longer than about 70 nm have readily identifiable long axes in dSTORM reconstructions. Such elongated filaments were readily identified. After validating our dSTORM imaging procedure, we utilized the methodology to interrogate the molecular structures underlying widefield Rad51 and Dmc1 foci *in vivo*.

Super-resolution light microscopy revealed that individual Rad51 (and Dmc1) foci observed by widefield microscopy are often composed of multiple distinct substructures. These Rad51 and Dmc1 “super-resolution foci”, hereafter referred to as sr foci, appeared to be paired or to be members of small clusters separated by less than 200 nm (Fig 4E and 4F). In our initial experiments, images generated by super-resolution analysis software displayed fine threads connecting close pairs of sr foci, but further analysis showed this feature of the images was artifactual (for details see Methods, S1 Text, and S2 Fig). Elimination of this artifact revealed pairs or clusters of distinct staining sr foci. In accord with the visual prevalence of pairing/clustering, the 100 nm peak in the Rad51-to-Rad51 (and Dmc1-to-Dmc1) nearest neighbor distribution was enriched relative to a randomly simulated distribution (Fig 4I and S3A Fig). Furthermore, 28% of Rad51 sr foci that were within 200 nm of at least one sr focus were within 200 nm of more than one sr focus. The nearest neighbor distributions of Rad51 sr foci and Dmc1 sr foci were similar in wild type diploids and *spo11* hypormorphic tetraploids (Fig 4I and S3A–S3C Fig). Importantly, like standard resolution foci, Rad51 and Dmc1 sr foci are predominantly *SPO11*-dependent (S4 Fig); the very faint *SPO11*-independent staining observed is likely to reflect binding of Rad51 and Dmc1 at non-DSB sites[44]. Artificially blurring dSTORM micrographs generates images that display the same nearest neighbor distributions as those obtained by the standard widefield method, indicating that the images produced by the two modalities are congruent (S5 Fig).

Comparison of widefield and dSTORM images demonstrates that standard resolution Rad51 (and Dmc1) foci are often composed of two or more constituent sr foci. In the context of paired standard resolution Rad51 foci (see Fig 1), each standard resolution focus contains an average of 1.53 ± 0.78 Rad51 sr foci (Fig 4L–4O). Additionally, nuclei with greater than four Rad51 sr foci were observed in *spo11* mutants heterozygous for the VDE cut site (S6 Fig). These observations suggest that more than one Rad51 (and more than one Dmc1) filament can occupy a single tract of ssDNA. It should also be noted that the dSTORM results indicate that many of the “single” foci seen by widefield, i.e. foci well-separated from their nearest neighbors, represent pairs or clusters of sr foci.

Like the ≤400 nm pairs observed with widefield microscopy, the Rad51-to-Rad51 (and Dmc1-to-Dmc1) nearest neighbor distributions obtained by dSTORM imaging were unaffected in strand exchange and synapsis mutants (Figs 4G and 4H and 4J and 4K and S3C and S3D). These results suggest that sr focus clusters form independently of strand exchange and persist until synapsis.

The Rad51 and Dmc1 structures observed with super-resolution microscopy are only slightly elongated (Fig 4E–4H and 4P). The longest dimension of the image of Rad51 and Dmc1 sr foci in wild type nuclei is 114 ± 29.9 and 119 ± 37.7 nm, respectively. Although these distances are greater than the resolution of the technique, the images of sr foci are only slightly elongated with aspect ratios of 1.27 ± 0.25 and 1.31 ± 0.23 (Fig 4P). The small size of sr foci suggests only a portion of the ssDNA formed by resection of a DSB is bound by Rad51 or Dmc1 (see Discussion).

**Fig 5:**
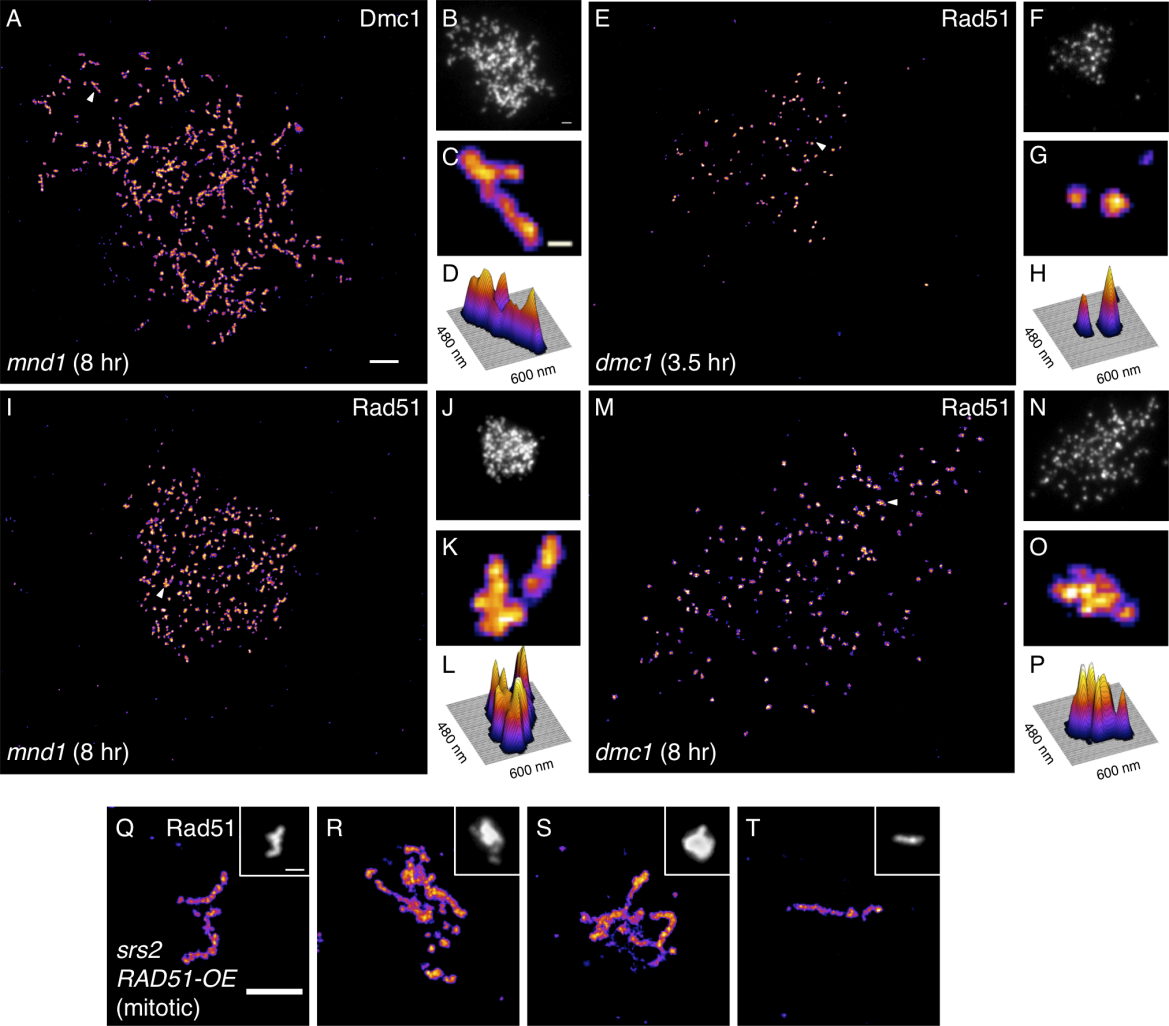
Elongated Dmc1 structures and higher order clustered Rad51 structures accumulate in strand exchange mutants at late times. (**A-D**) 8 hr *mnd1* nucleus stained for Dmc1. (**A**) dSTORM and (**B)** widefield micrographs each with 1 μm scale bars. (**C**) Magnified region from (A) indicated by arrowhead, highlighting elongated Dmc1 structure. Scale bar is 100 nm wide. (**D**) Surface plot of (C). (**E-H**) 3.5 hr *dmc1* nucleus stained for Rad51, highlighting a common pair (low order cluster) of sr foci. (**I-L**) 8 hr *mnd1* nucleus and (**M-P**) 8 hr *dmc1* nucleus stained for Rad51, highlighting higher order clusters of Rad51 sr foci. (**Q-T**) dSTORM micrographs of elongated Rad51 structures observed in spread nuclei of mitotic *srs2* mutants overexpressing Rad51. Scale bar is 1 μm.

We next asked if Rad51 and Dmc1 sr foci were altered when strand exchange is blocked. In wild type cells, Dmc1 foci are present and strand exchange occurs between 3 and 6 hours[8,13,16]; foci disappear and strand exchange is complete before 8 hours. If strand exchange is blocked in an *mnd1* or a *dmc1* mutant, DSB-associated ssDNA tracts become much longer than normal by 8 hours[8,13,34]. dSTORM microscopy of 3.5 hour *mnd1* nuclei revealed a punctate Dmc1staining pattern very similar to that seen in wild type (Fig 4H and 4P). However, the Dmc1 staining patterns seen in 8-hour *mnd1* nuclei were dramatically different; they contained numerous elongated structures with contour lengths often reaching 250 nm (Fig 5A–5D). This result indicates that elongation of Dmc1-containing structures is limited by Mnd1 function, likely because completion of Hop2-Mnd1-dependent strand exchange is associated with Dmc1 disassembly, as has been argued for Rad51 based on biochemical observations[45]. Importantly, no corresponding elongated Dmc1 structures have been observed in strand exchange-proficient cells (for example, Fig 4F and 4P). Like Dmc1 staining, little or no difference in the Rad51 staining was seen in either *mnd1* or *dmc1* mutants, as compared to wild type, at 3.5 hours (Figs 4G and 4P and 5E–5H). However, at 8 hours, *mnd1* nuclei frequently displayed clusters of about 3–7 poorly resolved Rad51 sr foci (Fig 5I–5L). These clusters lacked obvious elongated structure, in dramatic contrast to the fibrous staining patterns seen for Dmc1 using duplicate slides from the same cultures and time points (compare Fig 5A to 5I). Similar clustering of Rad51 sr foci was seen at 8 hours in *dmc1* cells (Fig 5M–5P), suggesting Dmc1 does not influence formation of Rad51 clusters when strand exchange is blocked. The Rad51 staining results are in agreement with previous low resolution studies showing Rad51 focus staining intensity increases with time in *dmc1* and *mnd1* mutants[16,33].

The observation that elongated Dmc1 structures are seen at 8 hours in *mnd1* mutants indicates that the small dimensions of sr foci in strand exchange-proficient cells truly reflect the dimensions of underlying structures in living cells, rather than a shortcoming of the chromosome spreading or staining methods. To further address this issue, we examined mitotic *srs2* mutant cells overexpressing Rad51. These mutant cells had previously been shown to form elongated structures by widefield microscopy[46]. In these cells, spread nuclei stained for Rad51 and imaged by dSTORM revealed filamentous structures with contour lengths of up to 1.5 μm (Fig 5Q–5T). Thus, we have observed highly elongated Dmc1 structures (Fig 5A–5D) and Rad51 structures (Fig 5Q–5T) *in vivo* with dSTORM imaging. Importantly, the widths of these elongated Rad51 and Dmc1 structures match the 70 nm width of the images of Rad51 filaments assembled *in vitro* and imaged under identical conditions (Fig 4A–4D). These results strongly suggest that the small Rad51 and Dmc1 sr foci observed in meiotic nuclei are indeed very short filaments. Furthermore, we can rule out several potential explanations for the size and arrangement of *in vivo* sr foci including disruption of filaments by the spreading procedure and incomplete antibody labeling. Finally, the clear contrast between elongated structures/filaments and clustered sr foci strengthens the conclusion that clustered sr foci associated with hyper-resected tracts of ssDNA represent distinct filamentous entities rather than an artifact of dSTORM imaging. In summary, dSTORM easily detects elongated Rad51 and Dmc1 filaments under our experimental conditions. Given this, we conclude that the Rad51 and Dmc1 sr foci observed in wild type meiosis represent underlying structures that are shorter than 40 protomers on average.

## Discussion

### A Single DSB End is Bound by Short, Often Multiple, Rad51- and Dmc1-Containing Structures

**Fig 6:**
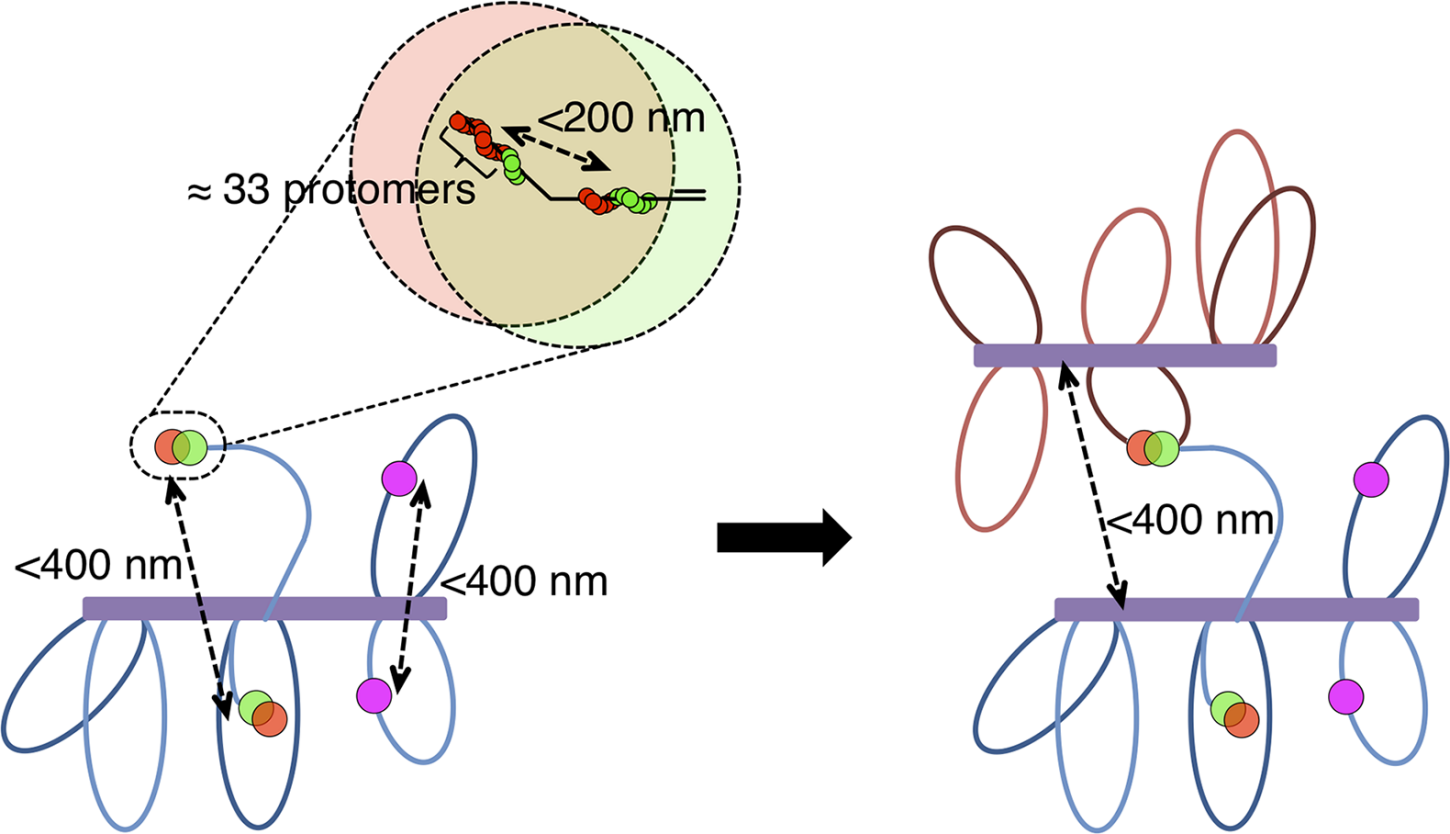
Recombinosome Model: Rad51 and Dmc1 each form short filaments on both spatially separated ends of a DSB. (Top) Short helical Rad51 and Dmc1 nucleoprotein homofilaments (green and red, respectively) form on adjacent segments of a single DSB-associated ssDNA tract. Each of these filaments is on the order of 100 nt or 33 protomers long and more than one Rad51-Dmc1 structure can form on a single tract of ssDNA. This single end of the DSB manifests itself cytologically as a side-by-side Rad51-Dmc1 co-focus (represented by the offset transparent red and green circles). (Left) Both Rad51 and Dmc1 similarly occupy the second end of the DSB. Prior to strand exchange, the two ends of the DSB are separated by 400 nm or less, resulting in focus pairing. The two ends of the DSB are both tethered to the axial element (purple box) by chromatin arms of variable length. Sister chromatids (pink circles) are often split in a DSB-independent manner. (Right) This architecture is maintained after strand exchange tethers the homolog at the predetermined distance of 400 nm or less.

Assuming that Rad51 and Dmc1 sr foci represent the DNA bound helical filaments that promote strand exchange *in vitro*, the sr foci we observed by dSTORM suggest that the Rad51 and Dmc1 filaments that promote recombination *in vivo* are extremely short and that more than one Rad51 and/or Dmc1 filament can form on the same ssDNA tract. Images of Rad51 and Dmc1 sr foci are only about 115 nm long. Since the diameter of Rad51 and Dmc1 filaments are known to be 10 nm [43], but sr foci are 70 nm wide, the images of sr foci observed with super-resolution microscopy following indirect immunostaining are likely about 60 nm larger in each dimension than the underlying protein complex. This difference can be accounted for by considering the size of the primary and secondary antibodies decorating the structure. Thus, assuming that RecA homolog structures represent the canonical nucleoprotein filaments[43], they are about 55 nm long. Given that Rad51 and Dmc1 filaments contain about 2 nt per nm[41,43,47], this corresponds to roughly 100 nt, 33 protomers of Rad51 (or Dmc1), and 5 turns of the helical nucleoprotein filament. This length estimate suggests that an individual Rad51 or Dmc1 filament typically occupies less than 15% of a typical 800 nt ssDNA tract [8,48]. An alternative, but in our view less likely, interpretation, is that RecA homologs coat a more substantial fraction of each ssDNA tract in a previously unknown compact configuration. Additionally, a significant fraction of the closely spaced (<200 nm apart) Rad51 sr foci likely represent loading of distinct filaments on the same tract of ssDNA as evidenced by detection of nuclei with more than 4 Rad51 sr foci in VDE cut site heterozygote nuclei and the accumulation of clustered Rad51 sr foci at late time points in strand exchange mutants. Although 100 nt filaments are quite small relative to those that have been studied in many biochemical experiments[49], both ensemble and single molecule experiments have shown that only 8 nt is sufficient for recognition of homology by RecA-like strand exchange proteins [50,51]. Thus, the size of the structures we observe is more than sufficient to promote efficient recombination. The finding that Rad51 and Dmc1 structures are small relative to the average length of ssDNA tracts is in agreement with previous observations indicating that Rad51 foci display offset colocalization with foci formed by the recombination proteins RPA and Rad52. These observations suggest that RPA and Rad52 can simultaneously occupy ssDNA segments adjacent to regions bound by Rad51 and Dmc1[52]. Furthermore, the lack of an inverse relationship between Rad51 and Dmc1 staining intensity in a single co-focus supports the hypothesis that ssDNA tracts are not completely bound by RecA homologs. All of these observations suggest that the protein composition and organization of a tract of ssDNA associated with a meiotic DSB is highly heterogeneous (Fig 6, top).

### Rad51 and Dmc1 Occupancy Does Not Functionally Differentiate the 1^st^ and 2^nd^ Ends of a DSB

The evidence presented here for loading of both Rad51 and Dmc1 on both DSB ends suggests that the two ends may be functionally identical with respect to homology search and strand exchange activity. The observed paired co-focus structure also suggests a simple molecular mechanism through which Rad51 could mediate Dmc1 assembly[2]. Binding of Rad51 to a ssDNA tract could enhance the efficiency of nearby Dmc1 filament initiation on that same tract [16,22,53,54]. Furthermore, although the two DSB ends engage the homolog in temporal succession[55], the first invading end need not be predetermined. Rather, the two ends of a DSB might both be released from the tethered loop complex in which they are formed (Fig 6, left) [56]. It is possible that both ends then compete to locate and invade a homolog chromatid with the “winner” maturing into the stable single end invasion intermediate[55]. Indeed, the presence of Dmc1—and thus its homology search and strand exchange activity—on both ends of a single DSB accounts for the existence of joint molecules connecting more than two chromatids[23]. These multichromatid joint molecules are not readily accounted for by the model positing loading of Rad51 and Dmc1 on opposite DSB ends, because the ability of Rad51 to form homology-dependent joint molecules is inhibited by Hed1 protein during meiotic prophase[11].

While our data argue that a large fraction of DSBs load both Rad51 and Dmc1 on both ends, a subset of structures in our micrographs do not fit into this class. At standard resolution, about 15% of Rad51 foci do not colocalize with Dmc1 foci (and vice versa), representing at least one ssDNA tract lacking Dmc1 (or Rad51). At super-resolution, about 21% of Rad51 sr foci lack a neighboring Rad51 sr focus within 400 nm, indicating a Rad51-coated ssDNA end unaccompanied by another Rad51-coated ssDNA tract within 400 nm. These results could be explained by a number of non-mutually exclusive possibilities. First, some focus pairs may be too close together to be resolved, even by dSTORM. Second, it is likely that a substantial fraction of structures formed by Rad51 and Dmc1 are too small to be detected by our methods. Consistent with this, focus staining intensities vary dramatically with some foci being only barely detectable above background. Third, the spread nuclei analyzed represent static snapshots of a possibly dynamic and undefined recombinosome assembly and disassembly process that ultimately passes through a stage where both Rad51 and Dmc1 occupy both DSB ends. Fourth, some DSBs may be processed by alternate pathways that do not require Rad51 and Dmc1 loading on both ends, as observed in a *rad51* mutant for example[13,22].

### The Two Ends of a Meiotic DSB Separate

Despite the proposed molecular symmetry, our results suggest that DSB ends can separate to distances of up to, and only rarely longer than, 400 nm. This distance is reminiscent of the conserved distance of 400 nm or less at which homologs are initially aligned[57]. Homolog alignment occurs at sites of nascent strand exchange intermediates—called axial association sites—prior to engagement of the second end of the DSB[36,38,58]. Thus, we interpret Rad51-Dmc1 co-foci paired at distances up to 400 nm as representing the structures responsible for the strand exchange-dependent component of homolog alignment (Fig 6, right). This cytological pattern of spatial separation between pairs of foci composed of recombination proteins Rad51 and Mer3, has previously been observed during leptotene/zygotene in *Zea mays* and *Sordaria macrospora*, respectively[58,59]. Yet, surprisingly, the paired Rad51-Dmc1 co-focus architecture depends on neither strand exchange nor synapsis in *S*. *cerevisiae*. Thus, we conclude that the two ends of a DSB are separated by up to 400 nm prior to and after strand exchange (Fig 6). In other words, we propose that the structure of a pre-strand exchange intermediate determines the distance at which homolog axes will be aligned.

What determines the distance at which the two ends of a DSB are separated? The variability up to 400 nm suggests that this length is not strictly determined by a fixed proteinacious scaffold, such as the Zip1 protein, which is responsible for the defined 100 nm spacing of lateral elements in the synaptonemal complex[60]. Instead, we propose that DSB formation within a chromatin loop[56], followed by release of the two flexible chromatin arms anchored at sites of sister chromatid cohesion along developing axial elements, allows the two DSB ends to separate as observed. The distribution of sister chromatid splitting distances rarely exceeds 600 nm, suggesting that chromatin loops are about 600 nm in length, similar to estimates of loop size obtained by electron microscopy[61]. If this loop size is correct, the lengths of the two released chromatin arms sum to about 600 nm, a distance in reasonable agreement with the separation distances detected for pairs of Rad51 or Dmc1 foci. We note that the DSB-independence of the sister chromatid splitting favors our hypothesis that the ends passively separate and does not provide support for the idea that meiotic recombination events involve local loss of sister chromatid cohesion or disassembly of chromatin to form a long homology-searching tentacle capable of searching the nuclear volume without associated movement of chromosome axes [62]. It is also in agreement with the alternative possibility that the searching entity is a chromatin “arm,” rarely longer than 400 nm, that extends from the axial element [62]. It is also important to note that although the ≤400 nm axis alignment distance is conserved in diverse organisms, meiotic chromatin loop size is not [57]. Thus, the similarity between focus separation distances and chromatin loop size could be unique to *S*. *cerevisiae*.

## Materials and Methods

### Strains

Tetraploid *spo11* hypomorphic strains were constructed by mating a *SPO11/spo11-Y135F* a/Δ diploid with a *spo11-Y135F/spo11-Y135F* α/Δ diploid[63]. The a/Δ and α/Δ diploids were obtained by gene targeting with DNA constructs designed to delete the *MATα* or the *MATa* locus, respectively. The strain with cytological landmarks in Fig 2 was constructed by combining the *HIS4*::*LEU2* DSB hotspot[55], a *tetO* array, a *lacO* array, YFP-TetR, and 3xHA-LacI via genetic crosses[64]. *lacO* and *tetO* arrays were inserted into *Chr III*, centromere proximal and distal to the *HIS4*::*LEU2* DSB hotspot, respectively, using the cloning-free method[65]. For the *spo11* VDE cut site heterozygote experiment in Fig 2, DKB 4571 was constructed by mating of YOC 3524 and YOC 3525[30], provided by the Ohya Lab. DKB 5369 was constructed by transformation of YOC 3524 and YOC 3525 with a PCR product designed to introduce an *mnd1* mutation followed by mating. See S1 Text for further details and S1 Table for genotypes.

### Meiosis and Cytology

Transfer to sporulation medium was used to induce synchronous meiotic cultures and preparation of spread chromosomes was previously described[16]. For experiments requiring visualization of LacI-3xHA, 4 mM PMSF was added to spheroplast suspensions and the solutions used for spreading. Rabbit anti-Rad51 (#159) and goat or guinea pig anti-Dmc1 (#189 or #174) antibodies were utilized at 1:1000 dilutions. Chicken anti-GFP (Invitrogen) was used at 1:1000, and mouse anti-HA (Santa Cruz) was used at 1:100. Alexa fluor 488 and 594 labeled secondary antibodies (Invitrogen) were used to stain Rad51 and Dmc1. Alexa fluor 647 and 750 labeled secondary antibodies (Invitrogen) were used to stain for GFP and HA. All secondary antibodies were used at 1:1000, except Alexa fluor 750 which was used at 1:100. For Fig 2A–2E, the goat anti-Zip1 antibody (Santa Cruz, sc-15632) was used at 1:1000 and stained with 1:1000 Alexa 594 secondary antibody. Images were acquired on a Zeiss Axiovision 4.6 microscope at 100X magnification and adjusted for brightness and contrast on ImageJ/FIJI software. For all two-color experiments, proper registration of image pairs obtained with different filter sets was confirmed using fluorescent beads (Molecular Probes, L-5241).

For dSTORM microscopy, spreads were stained with 1:1000 rabbit anti-Rad51 (or 1:1000 goat anti-Dmc1) and then with 1:1000 Alexa fluor 647 secondary. 0.05% Triton X-100 was included in the TBS washes to reduce background. Coverslips were mounted on a depression well slide filled with 10 mM MEA (prepared in PBS) and sealed with a two-part curable rubber product called “Body Double” (Smooth-On, Inc). Image sequences were acquired on a Leica SR GSD 3D microscope in 2D epifluorescence mode. Depletion with the 642 laser at 100% power was performed until the frame correlation dropped below 0.05, then acquisition commenced at 60% laser power. At least 25,000 frames were acquired. Images were reconstructed with the QuickPALM plugin[66] on FIJI using: an input pixel size of 100 nm, a reconstruction pixel size of 20 nm, a minimum SNR of 5.00, minimum symmetry of 0%, local threshold of 25%, maximum iterations per frame of 1000, and 50 threads. Also, a FWHM of 2 pixels was used to eliminate an artifact in which a large fraction of adjacent structures appear to be connected by a thin, sparsely populated thread (see S1 Text). This artifact is due to the almost simultaneous blinking of two adjacent fluorophores resulting in a “mis-called” event half way between two diffraction-limited blinks (S2 Fig). A 0.75 pixel Gaussian blur was applied to each micrograph before analysis.

For Fig 5Q–5T, Rad51 was over-expressed[46,67]. For Fig 4A–4D, *in vitro* Rad51 assembly reactions were performed as previously described[9] with the following exceptions. The assembly reaction included 0.5 nM 2.7 kb linear dsDNA (1.35 μM bp) generated by asymmetric PCR of pRS306 with one biotinylated primer, 1.6 μM Rad51, and 1 μM Hed1 to stabilize the filament[68]. The reaction was fixed with 3% PFA prepared in reaction buffer and then added to previously prepared coverslips coated with streptavidin by a modified version of a previously established protocol[69]. Slides were stained and imaged using dSTORM as described above.

### Analysis

Custom written ImageJ macros designated *4-spot macro* were used to generate the nearest neighbor distributions in Figs 1 and 2 and 3 and S1. Specifically, the (x,y) coordinates of focal centroids were determined manually with the ImageJ multipoint selection tool within the context of the macro. Note that Rad51 and Dmc1 foci were assumed to be diffraction limited spots (a valid assumption based on dSTORM micrographs). Thus, focus centroids as close as about 150 nm were often recognized as being distinct based on the fine appearance of staining structures (elongation vs perfectly circular focus, appearance of two maxima, etc), despite the fact that they were closer together than the resolution limit (around 250 nm). The nearest neighbor distributions were generated by coalescing output from the ImageJ macro using Excel. Simulated distributions were generated with custom written ImageJ macros (see S1 Text for details).

dSTORM reconstructions were scored in FIJI. The elliptical selection tool was fit to each observed sr focus. The (x,y) coordinates and various other descriptors of the ellipses were measured. Nearest neighbor distributions were determined in Excel workbooks, and simulations were performed in ImageJ as above.

For the Zip1 experiment in Fig 2, the (x,y) coordinates of both Rad51 and Zip1 foci were determined in early prophase nuclei. Nuclei were selected for having both Rad51 and Zip1 staining, but only nuclei with a completely punctate Zip1 staining pattern were chosen for analysis. Scoring and simulation of nearest neighbor positions were performed as above.

For the *spo11* VDE cut site heterozygote experiment in Fig 2, unselected nuclei were scored, but only focus-positive nuclei are included in the analysis. Single foci (category I) include Rad51-only, Dmc1-only, and Rad51-Dmc1 co-foci. Similarly, paired foci include all varieties of single foci, located within 1 μm of each other (category II). The “>2 Rad51 and/or Dmc1 foci” class (category III) includes structures where all of those foci are within 1 μm one another. The distant class (category IV) represents nuclei in which foci are separated by distances greater than 1 μm, but there are no more than 2 Rad51 or Dmc1 foci. Finally, the “>2 Rad51 and/or Dmc1 foci and distant” class (category V) consists of nuclei with two distinct cytological complexes separated by greater than 1 μm where the sum or Rad51 or Dmc1 foci is greater than two.

Scoring the cytological landmark experiment in Fig 2 required multiple levels of filtering. First, only nuclei displaying *lacO* and *tetO* arrays were scored for experiments involving both landmarks. Meiotic proteolysis resulted in a large fraction of nuclei without *lacO* spots. Also, rare nuclei with >2 *lacO* or *tetO* spots were excluded from analysis. Furthermore, only nuclei with one Dmc1 focus within 300 nm of the point between the closest *tetO* and *lacO* foci and 0–2 Dmc1 foci within 1 μm of that Dmc1 focus were analyzed. All analysis was performed with ImageJ software.

## Supporting Information

S1 Fig Paired Rad51-Dmc1 co-foci are not the result of focus crowding and the staining intensities of Rad51 and Dmc1 in each constituent co-focus are unrelated to the other co-focus. (**A-F)** Focus crowding does not account for pairing of Rad51 foci or pairing of Dmc1 foci. Observed (red) and simulated (blue) Rad51-Rad51 nearest neighbor distributions **(A,C,E)** and Dmc1-Dmc1 nearest neighbor distributions **(B,D,F)** in different subsets of foci from wild type *spo11* hypomorphic nuclei. **(A,B)** The raw, unfiltered set of nuclei replicated from Fig 1F and 1G for comparison. **(C,D)** Nearest neighbor distributions of low-density nuclei (<0.8 Rad51 or <0.8 Dmc1 foci per μm^2^ of nuclear area). **(E,F)** Nearest neighbor distributions of Rad51 or Dmc1 foci that are located in sparsely populated regions of the nucleus (exactly 1 Rad51 or Dmc1 focus within a 1 μm horizon of the focus). Micrographs from cultures 2.5 hours after meiotic induction. Sample sizes are 13,528 (A), 4,344 (C), and 1,624 (E) Rad51 foci and 13,230 (B), 4,251 (D), and 1,714 (F) Dmc1 foci. (**G-J**) Rad51 (or Dmc1) staining intensity in one co-focus is unrelated to the staining intensity of Rad51 (or Dmc1) in the other co-focus of a pair of co-foci. (**G**) Scatterplot displaying the brightness of a Rad51 focus vs. that of its associated Dmc1 focus, in 102 pairs of co-foci from *spo11* hypomorphic tetraploids at 2.5 hr time point. The brightness of a Rad51 (or Dmc1) focus is expressed as the percentage of total Rad51 (or Dmc1) signal in the Rad51 (or Dmc1) pair. Each individual point represents one scored pair of co-foci. Linear regression revealed a best-fit line (black line) with a slope of zero, indicating no relationship. (**H-J**) Examples of paired co-foci where the brighter Rad51 focus is associated with the brighter Dmc1 focus (H, correlated); both Rad51 and Dmc1 foci are roughly equally bright in both co-foci (I); and the brighter Rad51 focus is associated with the fainter Dmc1 focus (J, anti-correlated). If Rad51 and Dmc1 brightness were correlated a best-fit line would have a positive slope, while a negative slope would result if they were anti-correlated.
(TIFF)

S2 Fig Thread-like artifact that can result from post-acquisition localization determination with dSTORM. (**A-F**) A single nucleus imaged and/or reconstructed under different conditions. Imaging was performed under 100% 642 nm laser excitation (low density blinking); later 405 nm laser was added to increase the frequency of blinking (high density blinking). The ImageJ plugin QuickPALM was used to reconstruct micrographs using a stringent or relaxed threshold (2 or 4 pixels, respectively, input into QuickPALM as the “FWHM”) to localize events from raw image stacks. (**A**) Low-density blinking and stringent threshold (0.415 events/μm^2^/sec). (**B**) High-density blinking and stringent threshold (2.09 events/μm^2^/sec). (**C**) Low-density blinking and relaxed threshold (0.742 events/μm^2^/sec). (**D**) High-density blinking and relaxed threshold (2.95 events/μm^2^/sec). 33,378 and 8,375 frames were utilized for the low- and high-density reconstructions, respectively, resulting in about 51,500 “events” called in both frames (C) and (D). Arrowheads indicate the location of threads formed in (D). (**E,F**) Magnified version of boxed region in (C,D). (**G**) Statistical test for artifactual features. As the width cutoff in the image reconstruction algorithm is reduced, real features do not change in relative intensity (peaks near 100 nm and 400 nm in the line scan), but artifactual features due to multiple emitters decay (localizations near 250 nm). (**H**) Threads are the result of localizing an “event” in between two simultaneously fluorescing molecules located several hundred nanometers apart. One example “event” localized to the thread indicated in (F) is shown in frame 0 (relative frame numbers indicated in upper right). A diffraction-limited spot corresponding to a fluorophore in the bottom structure in (E,F) is fluorescing in frames -3 to 0. A separate diffraction-limited spot corresponding to a fluorophore in the top structure in (E,F) is fluorescing in frames 0 to +3. The simultaneous fluorescence of these nearby fluorophores in frame 0 results in mis-localizations that appear as a thread in between the two legitimate structures.
(TIFF)

S3 Fig Additional nearest neighbor distributions from dSTORM data sets. Nearest neighbor distributions for **(A)** Dmc1 sr foci in WT diploids, **(B)** Rad51 sr foci in *spo11* hypomorphic tetraploids, **(C)** Dmc1 sr foci in *spo11* hypomorphic tetraploids, and **(D)** Dmc1 sr foci in *mnd1 spo11* hypomorphic tetraploids. Sample sizes are 274 Dmc1 sr foci in 4 nuclei, 1084 Rad51 sr foci in 10 nuclei, 359 Dmc1 sr foci in 3 nuclei, and 592 Dmc1 sr foci in 6 nuclei, respectively.
(TIFF)

S4 Fig When observed by dSTORM, *SPO11*-independent Rad51 sr foci are small, faint, and less numerous than *SPO11*-dependent Rad51 structures. Micrographs of wild type **(A)**, *spo11* **(B,C)**, and *spo11* VDE cut site heterozygote **(D)** nuclei. The nuclei in **(B)** and **(C)** have very little and significant *SPO11*-independent Rad51 staining, respectively. Columns i) and ii) show the same dSTORM reconstruction, the latter displayed more brightly at the expense of signal saturation in some regions. Similarly, columns iii) and iv) show the same widefield micrograph of Rad51 staining displayed at two different brightness levels. Column v) shows the Zip1 staining pattern. Zip1 polycomplex served as a convenient means to locate nuclei lacking bright Rad51 staining patterns. In the micrograph of a *spo11* VDE cut site heterozygote shown in (D) the bright (VDE-dependent) and faint (VDE- and *SPO11*-independent) structures are readily distinguished. Micrographs from cultures 4 hours after meiotic induction. Scale bar is 1 μm wide.
(TIFF)

S5 Fig dSTORM and widefield micrographs are internally consistent. A single nucleus stained for Rad51 is imaged with **(A)** widefield microscopy and **(B)** dSTORM. The reconstructed dSTORM image is subjected to a **(C)** 6 pixel Gaussian blur, which approximates the transformation of the high resolution micrograph into the low resolution widefield micrograph. A small area (arrowhead) is magnified in insets at top right. Scale bar is 1 μm wide (or 200 nm wide in the inset). **(D)** Rad51-to-Rad51 nearest neighbor distributions under each of the conditions are plotted. Note that the peak of the distribution is around 100 nm for the dSTORM micrograph without blur and around 300 nm for either the widefield micrograph or the blurred dSTORM micrograph.
(TIFF)

S6 Fig dSTORM reveals additional sub-diffraction organization of Rad51 foci in *spo11* VDE cut site heterozygous strains. **(A-D)** Example dSTORM micrographs of Rad51 sr foci with corresponding widefield images inset at top right. Micrographs from cultures 4 hours after meiotic induction. All strains are *spo11* VDE cut site heterozygotes. **(A,B)** are otherwise wild type and **(C,D)** are *mnd1*. Scale bar is 1 μm wide. Note that there are more than four Rad51 sr foci revealed by dSTORM in each image and that these structures are not elongated.
(TIFF)

S1 Table Yeast strain table. (TIFF)

S1 Text Supplemental Materials. Supplemental Materials and Methods section includes further information about strain construction and image simulation. Supplemental Text section contains further discussion of the dSTORM reconstruction artifact described in the text.
(DOCX)

S1 Workbook Supplemental numerical data from histograms. (XLSX)
