# Supplementary figures and images for: Small Rad51 and Dmc1 Complexes Often Co-occupy Both Ends of a Meiotic DNA Double Strand Break
Source: PLoS Genet. 2015 Dec 31;11(12):e1005653. doi: 10.1371/journal.pgen.1005653 (PMC4697796; doi:10.1371/journal.pgen.1005653)

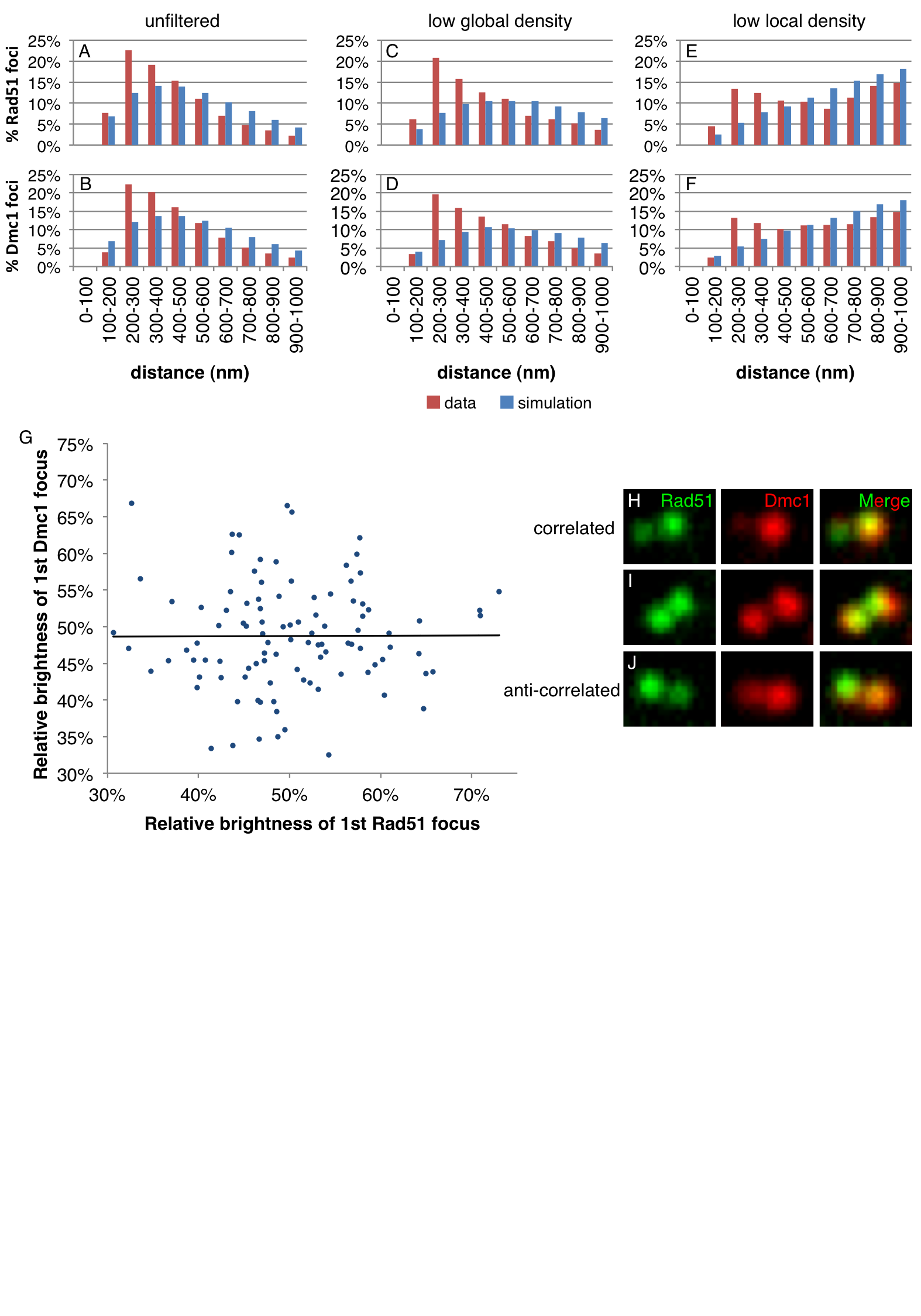

Supplement: S1 Fig — (A-F) Focus crowding does not account for pairing of Rad51 foci or pairing of Dmc1 foci. Observed (red) and simulated (blue) Rad51-Rad51 nearest neighbor distributions (A,C,E) and Dmc1-Dmc1 nearest neighbor distributions (B,D,F) in different subsets of foci from wild type spo11 hypomorphic nuclei. (A,B) The raw, unfiltered set of nuclei replicated from Fig 1F and 1G for comparison. (C,D) Nearest neighbor distributions of low-density nuclei (<0.8 Rad51 or <0.8 Dmc1 foci per μm2 of nuclear area). (E,F) Nearest neighbor distributions of Rad51 or Dmc1 foci that are located in sparsely populated regions of the nucleus (exactly 1 Rad51 or Dmc1 focus within a 1 μm horizon of the focus). Micrographs from cultures 2.5 hours after meiotic induction. Sample sizes are 13,528 (A), 4,344 (C), and 1,624 (E) Rad51 foci and 13,230 (B), 4,251 (D), and 1,714 (F) Dmc1 foci. (G-J) Rad51 (or Dmc1) staining intensity in one co-focus is unrelated to the staining intensity of Rad51 (or Dmc1) in the other co-focus of a pair of co-foci. (G) Scatterplot displaying the brightness of a Rad51 focus vs. that of its associated Dmc1 focus, in 102 pairs of co-foci from spo11 hypomorphic tetraploids at 2.5 hr time point. The brightness of a Rad51 (or Dmc1) focus is expressed as the percentage of total Rad51 (or Dmc1) signal in the Rad51 (or Dmc1) pair. Each individual point represents one scored pair of co-foci. Linear regression revealed a best-fit line (black line) with a slope of zero, indicating no relationship. (H-J) Examples of paired co-foci where the brighter Rad51 focus is associated with the brighter Dmc1 focus (H, correlated); both Rad51 and Dmc1 foci are roughly equally bright in both co-foci (I); and the brighter Rad51 focus is associated with the fainter Dmc1 focus (J, anti-correlated). If Rad51 and Dmc1 brightness were correlated a best-fit line would have a positive slope, while a negative slope would result if they were anti-correlated. (TIFF) [file pgen.1005653.s001.tiff]

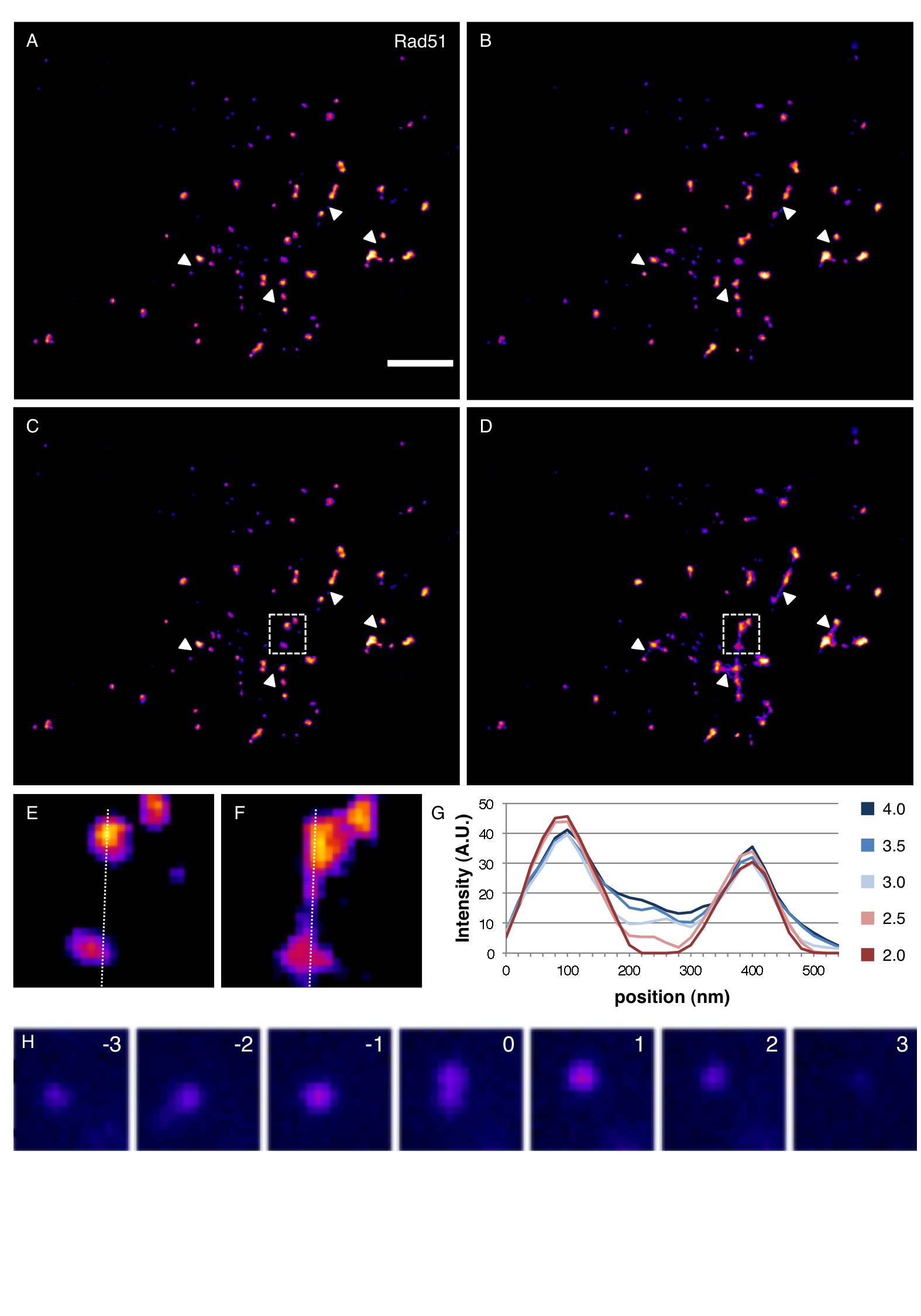

Supplement: S2 Fig — (A-F) A single nucleus imaged and/or reconstructed under different conditions. Imaging was performed under 100% 642 nm laser excitation (low density blinking); later 405 nm laser was added to increase the frequency of blinking (high density blinking). The ImageJ plugin QuickPALM was used to reconstruct micrographs using a stringent or relaxed threshold (2 or 4 pixels, respectively, input into QuickPALM as the “FWHM”) to localize events from raw image stacks. (A) Low-density blinking and stringent threshold (0.415 events/μm2/sec). (B) High-density blinking and stringent threshold (2.09 events/μm2/sec). (C) Low-density blinking and relaxed threshold (0.742 events/μm2/sec). (D) High-density blinking and relaxed threshold (2.95 events/μm2/sec). 33,378 and 8,375 frames were utilized for the low- and high-density reconstructions, respectively, resulting in about 51,500 “events” called in both frames (C) and (D). Arrowheads indicate the location of threads formed in (D). (E,F) Magnified version of boxed region in (C,D). (G) Statistical test for artifactual features. As the width cutoff in the image reconstruction algorithm is reduced, real features do not change in relative intensity (peaks near 100 nm and 400 nm in the line scan), but artifactual features due to multiple emitters decay (localizations near 250 nm). (H) Threads are the result of localizing an “event” in between two simultaneously fluorescing molecules located several hundred nanometers apart. One example “event” localized to the thread indicated in (F) is shown in frame 0 (relative frame numbers indicated in upper right). A diffraction-limited spot corresponding to a fluorophore in the bottom structure in (E,F) is fluorescing in frames -3 to 0. A separate diffraction-limited spot corresponding to a fluorophore in the top structure in (E,F) is fluorescing in frames 0 to +3. The simultaneous fluorescence of these nearby fluorophores in frame 0 results in mis-localizations that appear as a thread in between th [file pgen.1005653.s002.tiff]

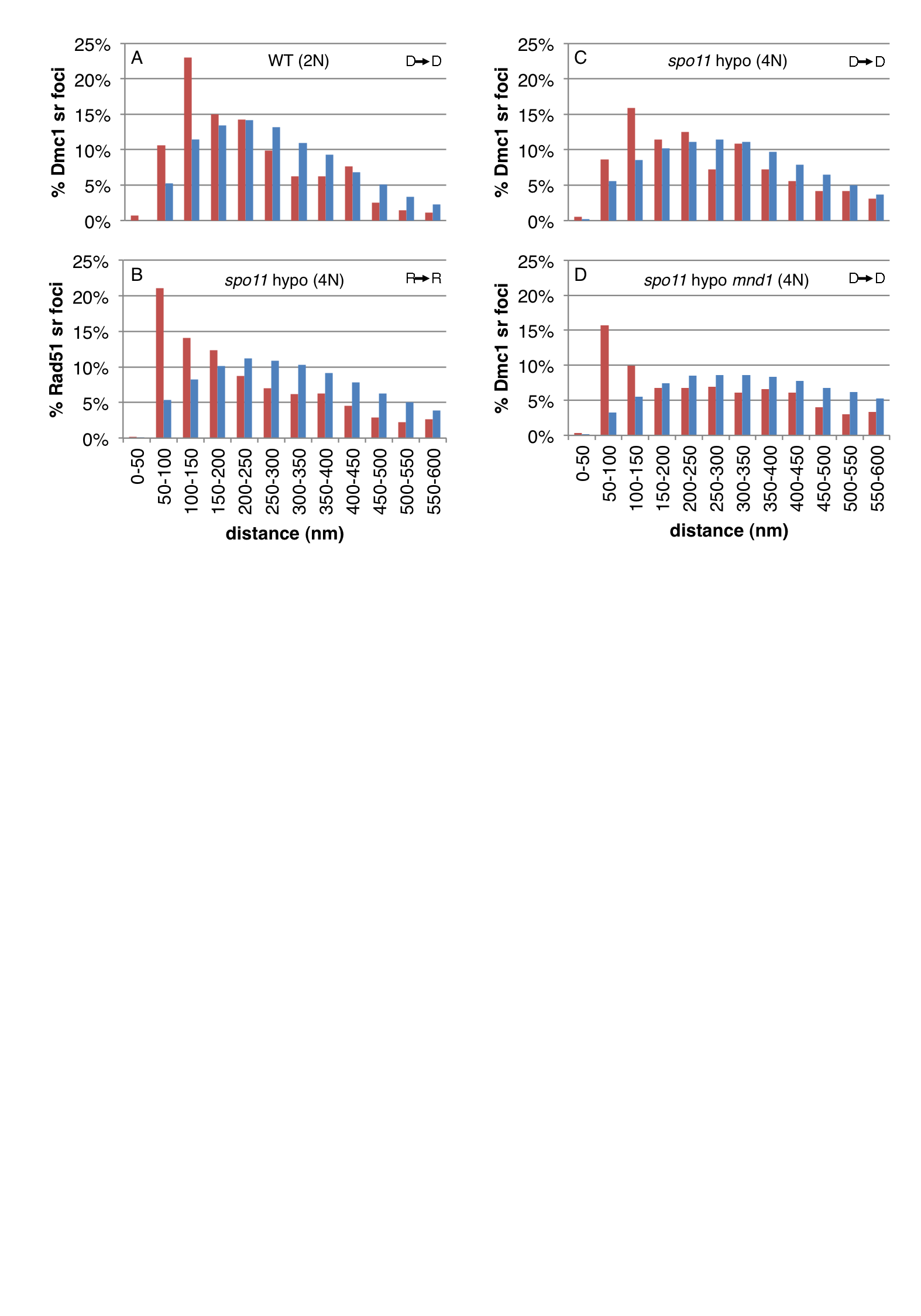

Supplement: S3 Fig — Nearest neighbor distributions for (A) Dmc1 sr foci in WT diploids, (B) Rad51 sr foci in spo11 hypomorphic tetraploids, (C) Dmc1 sr foci in spo11 hypomorphic tetraploids, and (D) Dmc1 sr foci in mnd1 spo11 hypomorphic tetraploids. Sample sizes are 274 Dmc1 sr foci in 4 nuclei, 1084 Rad51 sr foci in 10 nuclei, 359 Dmc1 sr foci in 3 nuclei, and 592 Dmc1 sr foci in 6 nuclei, respectively. (TIFF) [file pgen.1005653.s003.tiff]

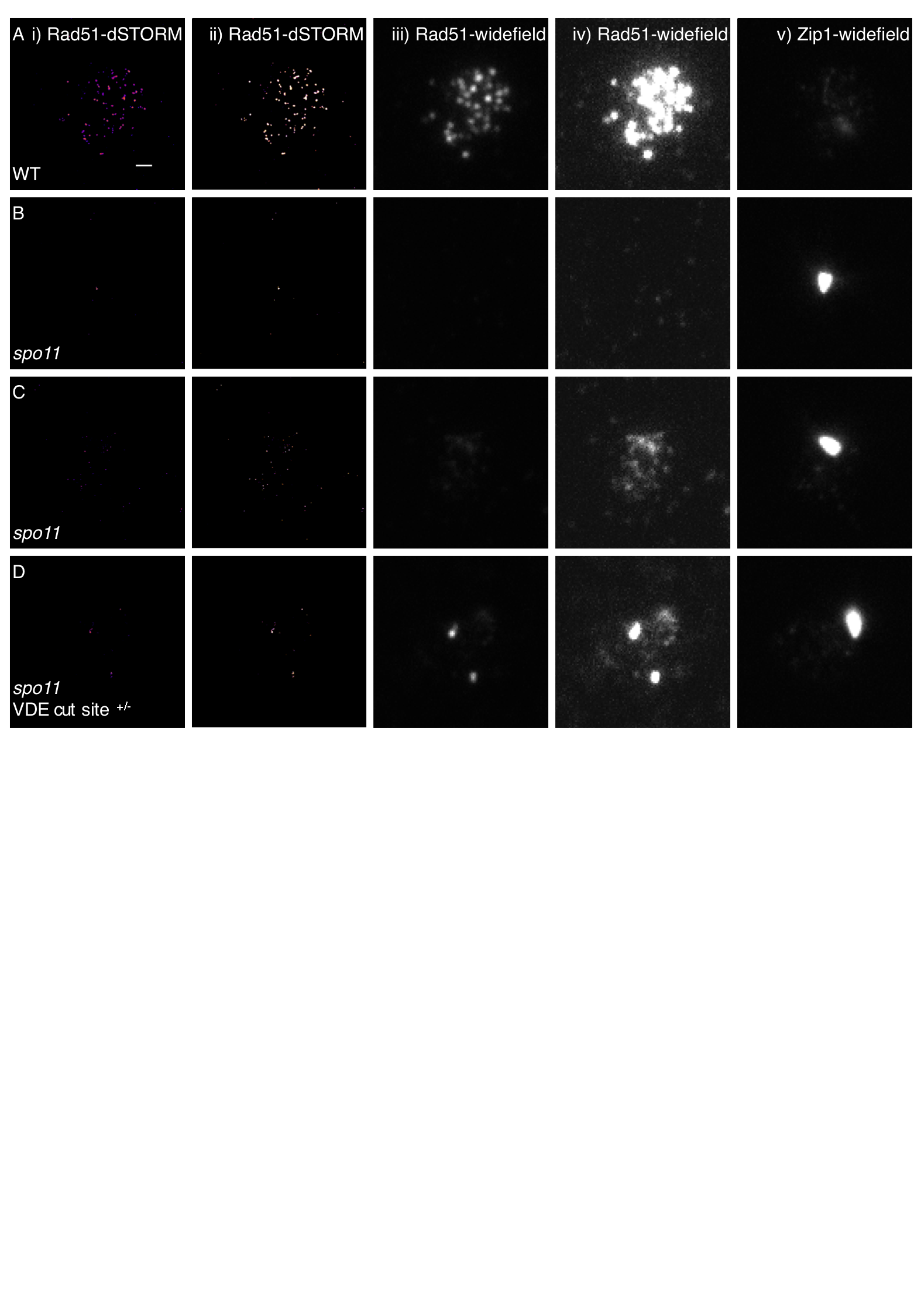

Supplement: S4 Fig — Micrographs of wild type (A), spo11 (B,C), and spo11 VDE cut site heterozygote (D) nuclei. The nuclei in (B) and (C) have very little and significant SPO11-independent Rad51 staining, respectively. Columns i) and ii) show the same dSTORM reconstruction, the latter displayed more brightly at the expense of signal saturation in some regions. Similarly, columns iii) and iv) show the same widefield micrograph of Rad51 staining displayed at two different brightness levels. Column v) shows the Zip1 staining pattern. Zip1 polycomplex served as a convenient means to locate nuclei lacking bright Rad51 staining patterns. In the micrograph of a spo11 VDE cut site heterozygote shown in (D) the bright (VDE-dependent) and faint (VDE- and SPO11-independent) structures are readily distinguished. Micrographs from cultures 4 hours after meiotic induction. Scale bar is 1 μm wide. (TIFF) [file pgen.1005653.s004.tiff]

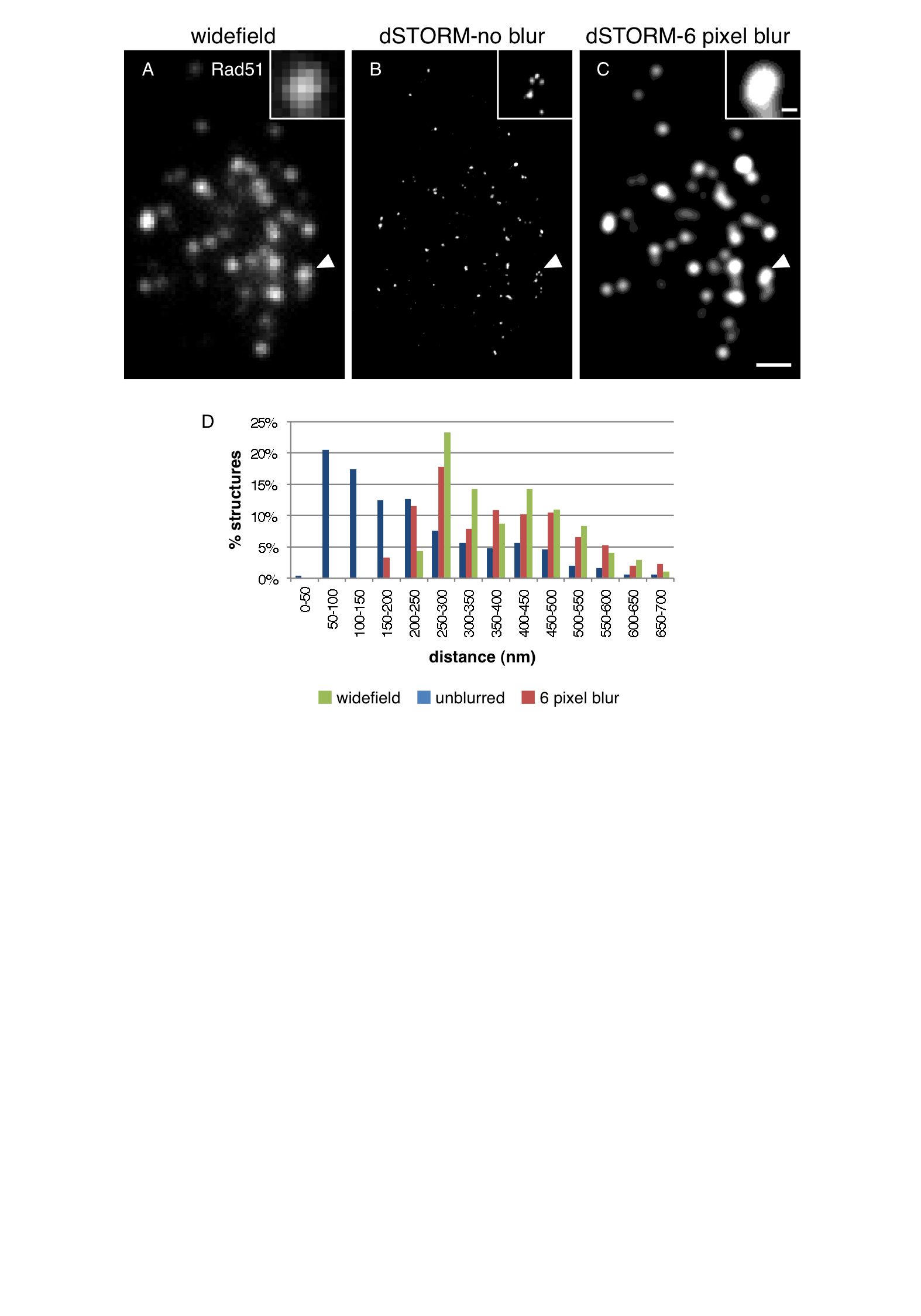

Supplement: S5 Fig — A single nucleus stained for Rad51 is imaged with (A) widefield microscopy and (B) dSTORM. The reconstructed dSTORM image is subjected to a (C) 6 pixel Gaussian blur, which approximates the transformation of the high resolution micrograph into the low resolution widefield micrograph. A small area (arrowhead) is magnified in insets at top right. Scale bar is 1 μm wide (or 200 nm wide in the inset). (D) Rad51-to-Rad51 nearest neighbor distributions under each of the conditions are plotted. Note that the peak of the distribution is around 100 nm for the dSTORM micrograph without blur and around 300 nm for either the widefield micrograph or the blurred dSTORM micrograph. (TIFF) [file pgen.1005653.s005.tiff]

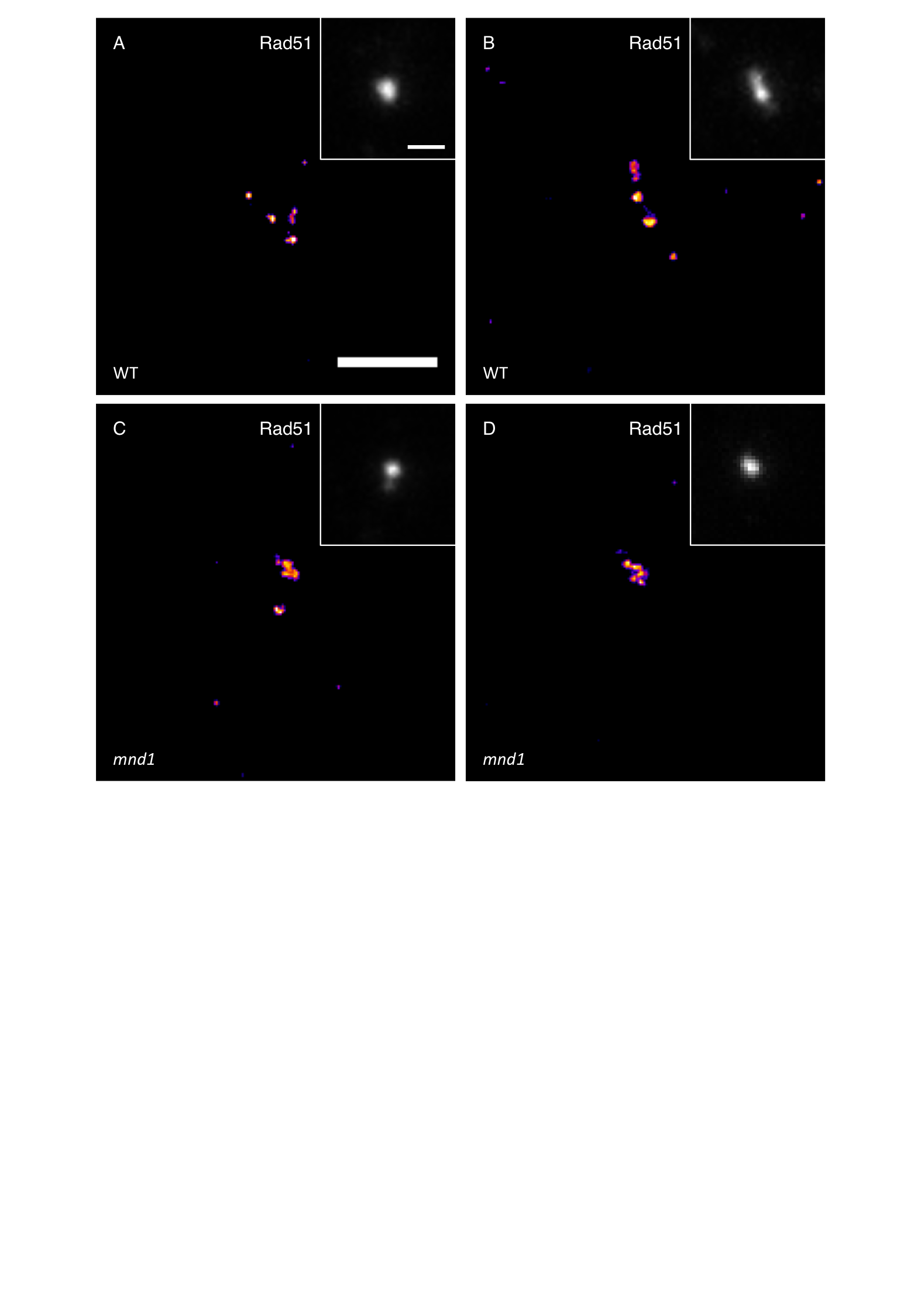

Supplement: S6 Fig — (A-D) Example dSTORM micrographs of Rad51 sr foci with corresponding widefield images inset at top right. Micrographs from cultures 4 hours after meiotic induction. All strains are spo11 VDE cut site heterozygotes. (A,B) are otherwise wild type and (C,D) are mnd1. Scale bar is 1 μm wide. Note that there are more than four Rad51 sr foci revealed by dSTORM in each image and that these structures are not elongated. (TIFF) [file pgen.1005653.s006.tiff]

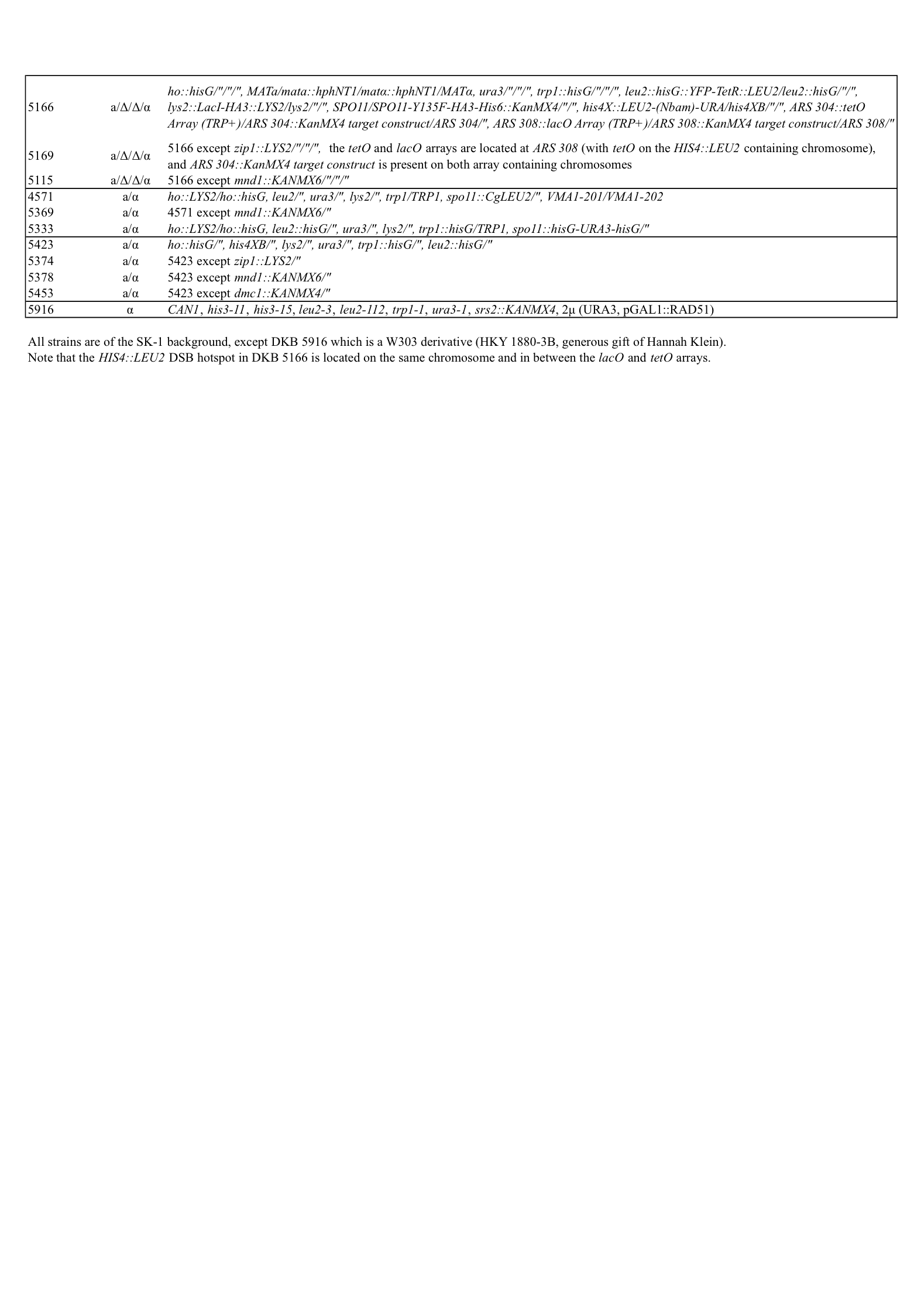

Supplement: S1 Table — (TIFF) [file pgen.1005653.s007.tiff]
