# Supplementary material for: Small Rad51 and Dmc1 Complexes Often Co-occupy Both Ends of a Meiotic DNA Double Strand Break
Source: PLoS Genet. 2015 Dec 31;11(12):e1005653. doi: 10.1371/journal.pgen.1005653 (PMC4697796; doi:10.1371/journal.pgen.1005653)
Supplement: S1 Text — Supplemental Materials and Methods section includes further information about strain construction and image simulation. Supplemental Text section contains further discussion of the dSTORM reconstruction artifact described in the text. (DOCX) [file pgen.1005653.s008.docx]

**Supplemental Materials and Methods**

**Strains**

*MATα* and *MATa* locus deletion constructs were generated by amplifying the *hphNT* fungicide resistance cassette from pNRB 598 with tailed primers to add homology arms. Specifically, 5’-cgaaacccagtttttgatttgaatgcgagataaactggtattcttccgtcccaaaaccttctca-3’ and either 5’-gttgcgcgaagtagtcccatattccgtgctgcattttgtccgcgtcagcgacatggaggc-3’ or 5’- catacccaaactcttacttgaagtggagtaatgccacatttctttgccatcagcgacatggaggc-3’ primers were used. *MATα /Δ* and *MATa /Δ* transformants were verified by mating type tests.

The *ARS304* and *ARS308* array-targeting constructs, pNRB 571 and pNRB 590 respectively, were constructed by tailed-PCR from pNRB 302 and TA-cloned. Specifically, primers 5’- agttaaattatccaattccaaattctagggacggtttccaagcggatgccgggagcagac-3’ and 5’- gttataattgtgtaaaagggcacagtcaatgagtagtagagtgagctgataccgctcgcc-3’ were used to construct pNRB 571. Primers 5’- ctaacacttgtcaaacagaatataaggattacttgtcttcagcggatgccgggagcagac-3’ and 5’- cgccatgccatgtttatgaaatgtataggtactgtactatgtgagctgataccgctcgcc-3’ were used in the construction of pNRB 590. Stains were first transformed with one of the targeting constructs. Second, linearized pSR10/pNRB 577 (277x *lacO* array/*TRP1^+^*) or pSR11/pNRB 578 (119x *tetO* array/*TRP1^+^*) was transformed, replacing the targeting module [1].

Fusion proteins designed to bind the *tetO* and *lacO* arrays were constructed by standard molecular methods. *YFP-TetR::LEU2* (pNRB 667) was constructed by overlap PCR and inserted into pNRB 567 via EcoRI and BamHI sites. After linearization of the plasmid with EcoRV, it was integrated at *LEU2*. *3xHA-LacI::LYS2* (pNRB 677) was constructed by overlap PCR and inserted into pNRB 592 via EcoRI and SphI sites. After linearization with StuI, it was integrated at *LYS2*.

The *mnd1* construct used to make the *spo11* VDE cut site heterozygotes was generated by PCR. Specifically, genomic DNA from DKB 5115 (*mnd1*) was amplified with 5’-cggctaatctgcaagctctatgcct-3’ and 5’-acctaagcctcttctcacgaaggca-3’.

**Analysis**

Simulated nearest neighbor distributions were generated from a custom-written ImageJ macro that independently chooses points within a defined two-dimensional space and measures their proximity to one another. Specifically, a matched list of nuclear areas and focus numbers (from experimentally observed data) were input. For each input nucleus, 10 nuclei were simulated (50 for dSTORM nuclei). The simulated nucleus was approximated by a circle of the same area. Simulated foci were placed iteratively and randomly within the nucleus. A simulated focus was rejected if it was located closer to another simulated focus than the closest inter-focus distance observed in the experimental data set (not just the single nucleus being simulated). The simulated nearest neighbor distances were coalesced in Excel for analysis and compared to the experimental distribution.

**Supplemental Text**

**Strategy for Reducing Artifacts in Super-Resolution Microscopy**

In preliminary analyses of super-resolution data sets, we observed that nearby pairs of punctate structures sometimes appeared to be connected by thin threads of localizations. We suspected that these thread-like objects might be artifacts of image analysis that do not reflect the underlying structure in the cell. Algorithms used to analyze dSTORM and other single-molecule localization data sets must make statistical calls to estimate which parts of the data set correspond to isolated fluorescent molecules. An important source of error is the common scenario where two nearby molecules are both emitting in the same frame. When the image of one of the emitting molecules is dim (for example, because it either switched into a dark state or into a fluorescent state in the middle of the frame), analysis software may treat the two molecules as single emitter, resulting in an artifactual localization that tends to lie along the line joining the two molecules. Although such mis-localization events have been described previously [2], their manifestation as thread-like structures has to our knowledge never been reported.

Image analysis algorithms use statistical tests to reject artifactual localizations by comparing data to an idealized point-spread function based on the expectation for a single fluorescent molecule. These algorithms contain tunable parameters that set thresholds for when a candidate localization will be rejected, e.g. the width or the ellipticity of the fluorescent spot. Here, we describe a general method that can be used to test for artifactual structures in a reconstructed image. The method involves creating several reconstructions by running a single-molecule localization algorithm on the same data set with various values of threshold parameters (e.g. spot width). These reconstructed images are then compared against each other. The intuitive expectation behind this procedure is that, while making a threshold parameter more stringent will cause some true single emitters to be rejected (type I errors), cases with multiple emitters will be correctly rejected more efficiently. Thus, one expects artifactual structures to fade relative to true structures as parameter stringency is increased (Fig. S2). Finally, we note that most artifactual localization problems originate with nearby emitters overlapping in time, so that any algorithm will become less susceptible to artifacts as the density of fluorophore switching events (per unit area per frame) decreases.

**Difficulties with Appropriately Setting the Spot Width Threshold**

When applying the above strategy, we found that in order to reduce the thread-like artifactual localizations to an acceptable level, we often had to set the spot width threshold to a value that seemed physically unreasonable. For example in QuickPALM, we had to set the FWHM (full width at half-maximum) parameter to 2 px to get satisfactory results, while the actual FWHM of isolated fluorescent spots in our images was 3.7 + 0.44 px corresponding to 370 nm (n=50 spots, measured manually).

We experienced a similar problem with multiple commercially available analysis packages, but chose to investigate the QuickPALM plug-in in depth because the source code for this algorithm is publicly available. The point-spread function of a diffraction-limited microscope projected onto one dimension can often be well approximated by a Gaussian:

$$I\left( x \right)= I_{\text{max}}e^{\frac{-x^{2}}{2\sigma^{2}}}$$

Where σ is the standard deviation. The FWHM of this Gaussian is:

$${FWHM}_{\text{Gauss}}=2\sqrt{2\text{ln}2}\sigma\approx2.355\sigma$$

The QuickPALM algorithm estimates spot width not by the standard deviation but by the mean absolute deviation, that is *MAD* = <|xI(x)|>.

$${MAD}_{\text{Gauss}}=\frac{2}{\sigma\sqrt{2\pi}}\int_{0}^{\infty} dxx e^{-\frac{x^{2}}{2\sigma^{2}}}=\frac{2}{\sqrt{2\pi}}\sigma$$

The QuickPALM algorithm implements a width cut-off by comparing 2.355⋅*MAD* to the user input FWHM. Since, for a Gaussian, *MAD* differs from σ by a factor 2/√(2π) ≈ 0.798, the result is that the effective FWHM being tested against will be ≈25% larger than the intended FWHM input by the user.

The simplest workaround for this discrepancy is to decrease the FWHM parameter in the QuickPALM input by a factor of ~0.8 relative to empirical estimates. Note that larger (uncorrected) values of the FWHM parameters result in substantial artifactual structures in our data (Fig. S2). We tested some other commercial super-resolution analysis packages and found that they produced the same artifactual thread-like structures (although we could not subject the reconstructions to the same kind of parameter sampling in those packages). It is therefore possible that similar discrepancies exist in other algorithms.

**Supplemental References**

1. Rohner, Sabine, Susan M Gasser, and Peter Meister. 2008. “Modules for Cloning-Free Chromatin Tagging in *Saccharomyces Cerevisae*..” *Yeast (Chichester, England)* 25 (3): 235–39. doi:10.1002/yea.1580.

2. van de Linde, Sebastian, Steve Wolter, Mike Heilemann, and Markus Sauer. 2010. “The Effect of Photoswitching Kinetics and Labeling Densities on Super-Resolution Fluorescence Imaging” 149 (4): 260–66. doi:10.1016/j.jbiotec.2010.02.010.
